# Protocatechuic acid prevents isoproterenol‐induced heart failure in mice by downregulating kynurenine‐3‐monooxygenase

**DOI:** 10.1111/jcmm.17869

**Published:** 2023-07-22

**Authors:** Liyan Bai, Xiongyi Han, Hae Jin Kee, Xiaonan He, Seong Hoon Kim, Mi Jin Jeon, Hongyan Zhou, Seong Min Jeong, Seung‐Jung Kee, Myung Ho Jeong

**Affiliations:** ^1^ Heart Research Center of Chonnam National University Hospital Gwangju Republic of Korea; ^2^ Hypertension Heart Failure Research Center Chonnam National University Hospital Gwangju Republic of Korea; ^3^ Emergency Critical Center, Beijing Anzhen Hospital Capital Medical University Beijing People's Republic of China; ^4^ Aerospace Center Hospital Peking University Aerospace School of Clinical Medicine Beijing People's Republic of China; ^5^ Department of Parasitology and Tropical Medicine Chonnam National University Medical School Hwasun Republic of Korea; ^6^ Department of Laboratory Medicine Chonnam National University Medical School and Hospital Gwangju Republic of Korea; ^7^ Department of Cardiology Chonnam National University Medical School Gwangju Republic of Korea

**Keywords:** cardiac hypertrophy, fibrosis, heart failure, kynurenine‐3‐monooxygenase, protocatechuic acid

## Abstract

Protocatechuic acid (3,4‐dihydroxybenzoic acid) prevents oxidative stress, inflammation and cardiac hypertrophy. This study aimed to investigate the therapeutic effects of protocatechuic acid in an isoproterenol‐induced heart failure mouse model and to identify the underlying mechanisms. To establish the heart failure model, C57BL/6NTac mice were given high‐dose isoproterenol (80 mg/kg body weight) for 14 days. Echocardiography revealed that protocatechuic acid reversed the isoproterenol‐induced downregulation of fractional shortening and ejection fraction. Protocatechuic acid attenuated cardiac hypertrophy as evidenced by the decreased heart‐weight‐to‐body‐weight ratio and the expression of Nppb. RNA sequencing analysis identified kynurenine‐3‐monooxygenase (Kmo) as a potential target of protocatechuic acid. Protocatechuic acid treatment or transfection with short‐interfering RNA against *Kmo* ameliorated transforming growth factor β1–induced upregulation of Kmo, Col1a1, Col1a2 and Fn1 in vivo or in neonatal rat cardiac fibroblasts. *Kmo* knockdown attenuated the isoproterenol‐induced increase in cardiomyocyte size, as well as Nppb and Col1a1 expression in H9c2 cells or primary neonatal rat cardiomyocytes. Moreover, protocatechuic acid attenuated Kmo overexpression–induced increases in Nppb mRNA levels. Protocatechuic acid or *Kmo* knockdown decreased isoproterenol‐induced ROS generation in vivo and in vitro. Thus, protocatechuic acid prevents heart failure by downregulating Kmo. Therefore, protocatechuic acid and Kmo constitute a potential novel therapeutic agent and target, respectively, against heart failure.

## 1 INTRODUCTION

Heart failure is a clinical syndrome caused by various heart diseases, including myocardial infarction, hypertension and aortic stenosis,^1^, ^2^, ^3^, ^4^ which are associated with left ventricular hypertrophy and fibrosis.^5^ Cardiac hypertrophy, which can be established using an osmotic minipump to infuse catecholamines (such as phenylephrine or isoproterenol), is defined as cardiac myocyte growth without an increased number of cells, resulting in an increased cardiac wall thickness and narrowed lumina of the cardiac chambers. The heart weight apparently increases in response to hypertrophic stimulation, whereas heart failure refers to a state wherein cardiac function deteriorates due to cardiac muscle weakening and luminal enlargement. The successful establishment of an isoproterenol‐induced heart failure model is dependent on the mouse species and the isoproterenol concentration.^6^ Both transverse aortic constriction (TAC) and infusions of isoproterenol are used to establish animal models of heart failure characterized by a reduced ejection fraction, which occurs when the heart does not pump efficiently and causes lung oedema. Cardiac fibrosis is characterized by collagen deposition in the myocardial interstitium and perivascular regions,^7^ which leads to heart failure. Therefore, the suppression of cardiac fibrosis is a potential therapeutic strategy for preventing progression to heart failure.

Hydroxybenzoic acid isomers prevent inflammation, cancer, oxidation, hypertension, cardiac hypertrophy and heart failure. For example, gallic acid (3,4,5‐trihydroxybenzoic acid), gentisic acid (2,5‐dihydroxybenzoic acid) and protocatechuic acid (3,4‐dihydroxybenzoic acid) prevent cardiac hypertrophy, fibrosis and hypertension, as reported recently.^8^, ^9^, ^10^, ^11^, ^12^, ^13^ Additionally, gallic acid and gentisic acid alleviate pressure overload–induced heart failure or angiotensin II–induced atrial fibrillation.^14^, ^15^, ^16^, ^17^ The structures of protocatechuic acid and gallic acid are similar; protocatechuic acid is present in vegetables, fruits and grains (purple rice bran)^18^ and exerts many beneficial anticancer effects,^19^ inflammation, fibrosis,^20^ oxidation,^21^ microbial activity and thrombosis.^22^ In a recent study, protocatechuic acid was found to attenuate cardiac hypertrophy in isoproterenol‐infused mice.^10^ Protocatechuic acid–mediated prevention of fibrosis may provide a basis for improving the pathology of heart failure.^11^, ^20^ Fibrosis is a very critical factor in heart failure and is the final stage of most cardiovascular diseases. Heart failure is emerging as an important syndrome and is associated with a high hospitalization‐related burden.^23^ However, the effects of protocatechuic acid on heart failure and its underlying mechanisms have not been elucidated. In our study, we aimed to demonstrate that protocatechuic acid can be a new drug for heart failure treatment.

Kynurenine‐3‐monooxygenase (Kmo), which is a nicotinamide adenine dinucleotide phosphate–dependent flavin monooxygenase, catalyses the conversion of kynurenine to 3‐hydroxykynurenine^24^ and mediates the catabolic step of the kynurenine pathway, which is the crucial route of tryptophan catabolism. Cardiovascular conditions, such as heart failure, are positively correlated with serum levels of aberrant kynurenine catabolites, including kynurenine, 3‐hydroxykynurenine and quinolinic acid.^25^, ^26^, ^27^ Based on Kmo expression profile information determined using RNA sequencing data, we hypothesized that Kmo plays an important role in the pathogenesis of heart failure. In this study, using Kmo knockdown and Kmo overexpression in primary neonatal cardiomyocytes and fibroblasts, we found that Kmo regulated cardiac hypertrophy and fibrosis.

This study was conducted to investigate the therapeutic effects of protocatechuic acid in an isoproterenol‐induced heart failure mouse model and to reveal the underlying pathogenetic mechanisms.

## 2 MATERIALS AND METHODS

### 2.1 Reagents

Protocatechuic acid (cat no. 37580, lot no. BCCF0948), isoproterenol (cat no. I5627, lot no. BCCC9596) and anti‐sarcomeric actin (cat no. A2172, lot no. 128M4892V) antibody were obtained from Sigma‐Aldrich. Anti‐Actb (cat no. sc‐47778, lot no. K2222), anti‐Fn1 (cat no. sc‐59826, lot no. F1814), anti‐Col1a1 (cat no. sc‐293182, lot no. H2619) and anti‐BNP (Nppb, sc‐271185, lot no. L3119) antibodies were purchased from Santa Cruz Biotechnology. Anti‐Kmo antibodies (cat no. MAB8050‐100, lot no. CMPW0119101) were purchased from Bio‐Techne. Alexa Fluor 488 phalloidin (cat no. A12379, lot no. 2129460) was obtained from Invitrogen. Anti‐Col 3 (ab7778) antibody was purchased from Abcam. Anti‐Smad3 (9523T, lot no. 7) and anti‐Smad4 (3845T, lot no. 3) were purchased from Cell Signaling.

### 2.2 Establishment of an animal model of heart failure

A heart failure model was established by administering high‐dose isoproterenol to male C57BL/6NTac mice; with a modified version of a previously reported protocol.^6^ All animal procedures were approved by the Animal Experimental Committee of Chonnam National University Medical School (CNUHIACUC‐22007) and performed according to the Guide for the Care and Use of Laboratory Animals (US National Institutes of Health Publications, 8th edition, 2011).

To induce heart failure, 7‐week‐old male C57BL/6NTac mice were anaesthetised with ketamine (100 mg/kg body weight) and xylazine (5 mg/kg body weight). Next, an osmotic minipump was implanted in the mice to infuse isoproterenol (80 mg/kg body weight/day) for 14 days. The animals were divided into the following four groups (*n* = 8 per group): sham + dimethyl sulfoxide (DMSO); sham + protocatechuic acid (100 mg/kg body weight/day); isoproterenol + DMSO; and isoproterenol + protocatechuic acid. Mice in the isoproterenol + protocatechuic acid group received daily intramuscular injections of protocatechuic acid from Day 6 of isoproterenol administration for 9 days. The dosage of protocatechuic acid (100 mg/kg body weight/day) was chosen based on the tolerable and safe concentration identified in previous studies.^10^, ^18^, ^28^

### 2.3 Echocardiography

Before echocardiography, mice were anaesthetised via intraperitoneal injection of tribromoethanol (avertin; 114 mg/kg). Echocardiography was performed using the Vivid S5 system model with a 13‐MHz linear array transducer (GE Healthcare), as described previously.^10^ M‐mode images and parameters were acquired from the short‐axis view of the left ventricle at the level of the papillary muscles. The cardiac parameters were measured on Days 5 and 14 of isoproterenol administration.

### 2.4 Histological analysis and picrosirius red staining

Cardiac tissues were fixed with 4% paraformaldehyde, rinsed with tap water and embedded in paraffin. The paraffin‐embedded tissues were sectioned to a thickness of 3 μm. For haematoxylin and eosin (H&E) staining, the sections were deparaffinized with xylene and hydrated in a descending ethanol series (100%–70%). The sections were washed with tap water and stained with haematoxylin for 4 min and eosin, followed by phloxine for 1 min. Next, the sections were dehydrated with ethanol, cleared with xylene and mounted for observation.

For picrosirius red staining, the paraffin‐embedded sections were deparaffinized with xylene overnight, dehydrated and hydrated with distilled water. The sections were then incubated with a drop of picrosirius red for 60 min. Next, the sections were quickly rinsed twice with acetic acid solution, rinsed with ethanol, sequentially treated with ethanol and xylene and mounted for observation. The tissue images were captured using a microscope (Nikon Eclipse 80*i* microscope, Nikon).

### 2.5 Quantitative real‐time polymerase chain reaction (qRT‐PCR)

Total RNA was extracted from the cardiac tissues using TRIzol reagent (Invitrogen), according to the manufacturer's instructions. To quantify the RNA concentration, the absorbance of the sample was measured at 260 nm using an ACTGene spectrophotometer (ASP‐2680). The isolated RNA was reverse transcribed into complementary DNA (cDNA) using TOPscript RT DryMIX (Enzynomics). The SYBR green PCR kit (Enzynomics) and specific primers were used to conduct qRT‐PCR analysis. The relative mRNA levels were calculated using the 2^−ΔΔCt^ method. The primers used in the qRT‐PCR analysis are shown in Table 1.

**TABLE 1:** Primers used in quantitative real‐time polymerase chain reaction analysis.

Genes	Primer sequence (5′–3′)
*Gapdh* (*rat*)	F: AACCCATCACCATCTTCCAGGAGC R: ATGGACTGTGGTCATGAGCCCTTC
*Nppb* (*rat*)	F: GACGGGCTGAGGTTGTTTTA R: ACTGTGGCAAGTTTGTGCTG
*Col1a1* (*rat*)	F: ACCCCAAGGAGAAGAAGCAT R: AGGTTGCCAGTCTGTTGGTC
*Cysltr1* (*rat*)	F: GCCTCACCACCTATGCCTTA R: CCTGTGGAGGCTCAAAACAT
*Kmo* (*rat*)	F: GAGTCCTACCCCAATGCAAA R: TGTAATCAAAACGGGGCTTC
*Lefty1 (rat)*	F: CAAGGACTATGGGGCTCAAG R: GGGCAAGGAAGTCATCTCTG
*Fn1* (*rat*)	F: AGCAAATCGTGCAGCCTCCG R: CCCCCTTCATGGCAGCGATT
*Smad3* (*rat*)	F: AGTTGGACGAGCTGGAGAAG R: TGTTGAAGGCGAACTCACAG
*Smad4* (*rat*)	F: TCAGCCAGCTACTTACCACC R: TGGAAATGGGAGGCTGGAAT
*Gapdh* (*mouse*)	F: GCATGGCCTTCCGTGTTCCT R: CCCTGTTGCTGTAGCCGTATTCAT
*Nppb* (*mouse*)	F: CTGAAGGTGCTGTCCCAGAT R: GTTCTTTTGTGAGGCCTTGG
*Col1a1* (*mouse*)	F: GAGCGGAGAGTACTGGATCG R: GCTTCTTTTCCTTGGGGTTC
*Fn1* (*mouse*)	F: GATGCACCGATTGTCAACAG R: TGATCAGCATGGACCACTTC
*Lefty1* (*mouse*)	F: AGCTCAAGGCAATTGTGACC R: TCATCTCTGAGGCGACACAC
*Tnfrsf9* (*mouse*)	F: TGGTGAGCTTCTCTCCCAGT R: CCTAGTGCTTCTCGGTTTCG
*Mmp12* (*mouse*)	F: CTGGTTCTTCTGGTGGAAGC R: ATGCTCCTGGGATAGTGTGG
*Enkur* (*mouse*)	F: ATGGACTCACCCTGCACTTC R: TCCTTGGGGGAAGGTATCTC
*Spp1* (*mouse*)	F: CCCGGTGAAAGTGACTGATT R: CCATCGTCATCATCATCGTC
*Pamr1* (*mouse*)	F: TGCAGCTCCTTCTCATCTCA R: GGATGGTGTAACCCACCACT
*Cfap61* (*mouse*)	F: GTACACCGGCACTGTTCCTT R: CATGCCAATGTTGAGGAGTG
*Cysltr1* (*mouse*)	F: CCTCACCACCTATGCCTTGT R: GTTCTGTGGAGGCTCAAAGC
*Kmo* (*mouse*)	F: AGGGGAAAAGAGTGGCTGTT R: AGGGCCAAGTTAATGCTCCT
*Ubxn10* (*mouse*)	F: TGCAACGGTCTCAACATCTC R: ACAGACCTGCGTTCTGGAGT
*Timp1* (*mouse*)	F: CTTCTTGGTTCCCTGGCGTA R: AAGTGACGGCTCTGGTAGTC
*Lgals3* (*mouse*)	F: ACCCAACGCAAACAGGATTG R: TTGACCGCAACCTTGAAGTG
*Egr3* (*mouse*)	F: TTCGCTTTCGACTCTCCTTC R: ATACATGGCCTCCACGTCTC
*Smad3* (*mouse*)	F: CACAGCCACCATGAATTACG R: GTGTTCTCGGGAATGGAATG
*Smad4* (*mouse*)	F: TCGATTCAAACCATCCAACA R: GCCCTGAAGCTATCTGCAAC
*Tnfα* (*mouse*)	F: AAGTCAACCTCCTCTCTGCC R: ACACCCATTCCCTTCACAGA
*Il‐1β* (*mouse*)	F: ACTCATTGTGGCTGTGGAGA R: TTGTTCATCTCGGAGCCTGT

### 2.6 Western blotting

Total proteins were extracted from the cardiac tissues or cardiac fibroblasts using radioimmunoprecipitation assay lysis buffer (150 mM NaCl, 1% Triton X‐100, 1% sodium deoxycholate, 50 mM Tris–HCl [pH 7.5], 2 mM ethylenediaminetetraacetic acid, 1 mM phenylmethylsulfonyl fluoride, 1 mM dithiothreitol, 1 mM Na_3_VO_4_ and 5 mM NaF) supplemented with a protease inhibitor cocktail (Calbiochem/EMD Millipore). The protein concentration was determined using the Pierce bicinchoninic acid protein assay kit (Thermo Fisher Scientific). Equal amounts (40 μg) of proteins were subjected to sodium dodecyl sulfate‐polyacrylamide gel electrophoresis using a 10% gel. The resolved proteins were transferred onto a polyvinylidene difluoride membrane (pore size = 0.45 μm), and the membrane was blocked with 5% skim milk and incubated with the primary antibodies (1:1000) at 4°C overnight. Next, the membrane was washed three times with Tris‐buffered saline containing Tween 20 buffer (20 mM Tris, 200 mM NaCl and 0.04% Tween 20) to remove unbound proteins. It was then incubated with anti‐rabbit or anti‐mouse horseradish peroxidase–conjugated secondary antibodies (1:5000) for 1 h at 25°C. Immunoreactive signals were visualized using Immobilon Western blotting detection reagents (EMD Millipore), and the protein band intensities were quantified using ImageJ (National Institutes of Health).

### 2.7 H9c2 cell culture

H9c2 cardiomyoblasts were purchased from the Korean Cell Line Bank and cultured in Dulbecco's modified Eagle's medium (DMEM)/high glucose (4500 mg/L) containing 10% foetal bovine serum (FBS), 100 U/mL penicillin and 100 μg/mL streptomycin in a 5% CO_2_ incubator at 37°C. Cells were passaged every 2 or 3 days.

### 2.8 Primary rat cardiac fibroblast culture

Primary rat cardiac fibroblasts were isolated from the cardiac tissues of 1‐ to 2‐day‐old rat pups, as described previously.^15^ The atrium was excised, and the ventricles were finely chopped using sterile scissors. The samples were incubated in 1× ADS buffer (116 mM NaCl, 20 mM HEPES, 10 mM NaH_2_PO_4_, 5.5 mM glucose, 5 mM KCl and 0.8 mM MgSO_4_) containing 0.1% collagenase type II on a shaker at 120 rpm for 2 h. To neutralize the collagenase activity, an equal amount of DMEM containing 20% FBS was added to the samples. The samples were then centrifuged, and the cell pellets were resuspended in DMEM containing 10% FBS. Cells that were passaged twice were used for the experiment. To investigate the effect of protocatechuic acid on fibrosis, rat cardiac fibroblasts were treated with protocatechuic acid 1 h before treatment with transforming growth factor (TGF)‐β1 (10 ng/mL).

### 2.9 Primary neonatal rat cardiomyocyte culture

Primary neonatal rat cardiomyocytes were isolated from the cardiac tissues of 1‐ to 2‐day‐old rat pups, as described previously.^8^ The ventricles were minced and digested with 0.1% collagenase type II and pancreatin. To complete tissue dissociation, FBS was added to stop enzymatic digestion and centrifuged at 1200 rpm for 5 min. Cell pellets were resuspended, and fibroblasts were allowed to adhere for 1 h in 100 mm dishes. Supernatant cells were centrifuged at 1200 rpm for 5 min, and the cells were subjected to Percoll gradients, with centrifugation at 3000 rpm for 30 min. The Percoll layer cells were washed using 1× ADS buffer and seeded in 8‐well plates or 12‐well plates. Cells were used for cell size and Western blot analyses.

### 2.10 Cell size and sarcomeric actin organization

Rat neonatal cardiomyocytes were plated on an 8‐well chamber, and immunocytochemistry was performed as described previously.^8^ The cells were transfected with si‐control or si‐Kmo (100 nM) for 48 h and serum‐starved overnight. The cells were incubated with isoproterenol (10 μM) for 48 h, fixed with 2% paraformaldehyde for 30 min and permeabilized with 0.1% Triton‐X 100. The cells were blocked with 3% goat serum for 1 h and incubated with sarcomeric α‐actin (1:200) overnight at 4°C. Then, secondary antibody (Alexa Fluor 568 goat anti‐mouse IgG) was added, and antifade reagent (including DAPI) was used to detect nuclei. The cell area was measured using NIS Elements Software (Nikon).

### 2.11 RNA sequencing

A library was independently prepared with 0.5 μg of total RNA for each sample using the Illumina TruSeq Stranded Total RNA Library Prep Gold kit (Illumina, Inc.). The ribosomal RNA in the sample was removed using the Ribo‐Zero rRNA Removal Kit (Human/Mouse/Rat Gold) (Illumina, Inc.). The remaining RNA was fragmented using divalent cations under elevated temperature conditions. The cleaved RNA fragments were reverse‐transcribed into first‐strand cDNA using SuperScript II reverse transcriptase. Second‐strand cDNA was synthesized using DNA polymerase I, RNase H and dUTP and thereafter ligated with adapters. The products were then purified and PCR‐amplified to generate the final cDNA library. The libraries were quantified using KAPA library quantification kits for Illumina sequencing platforms according to the qPCR Quantification Protocol Guide (Kapa Biosystems, #KK4864) and qualified using the TapeStation D1000 ScreenTape (Agilent Technologies, #5067–5582). Indexed libraries were subjected to paired‐end (2 × 100 bp) sequencing using Illumina NovaSeq (Illumina, Inc.) at Macrogen Inc. The datasets presented in this study can be found in online repositories (DNA Data Bank of Japan [DDBJ] with accession number DRA014580).

### 2.12 siRNA transfection

H9c2 cells were transfected with si‐control, si‐Cysltr1, si‐Kmo or si‐Lefty1 (100 nM, Bioneer) for 48 h. RNA was extracted from the transfected cells using TRIzol reagent. To investigate the effect of Kmo on cardiac fibrosis, both si‐control–transfected and si‐Kmo–transfected cardiac fibroblasts were treated with vehicle or TGF‐β1 (10 ng/mL). To investigate whether knockdown of Kmo affected the cell size and cardiac hypertrophy of primary neonatal rat cardiomyocytes, si‐control– and si‐Kmo–transfected cells were treated with vehicle or isoproterenol (10 μM).

### 2.13 Overexpression transfection

H9c2 cells or rat cardiac fibroblasts were seeded in 12‐well plates and transfected with *pCMV‐SPORT6* or *pCMV‐SPORT6‐mouse Kmo* (1000 ng/well) using Lipofectamine 3000 transfection reagents according to the manufacturer's instructions. Two days later, transfected cells were treated with or without protocatechuic acid for 9 h. The *pCMV‐SPORT6‐mouse Kmo* clone was purchased from the Korea Human Gene Bank, Medical Genomics Research Center, KRIBB.

### 2.14 Reactive oxygen species (ROS) production

Dihydroethidium (DHE) staining was performed to quantify the cellular or tissue ROS. H9c2 cells were seeded in 8‐well chambers and serum‐starved (0.1% FBS‐DMEM) 24 h. Cells were incubated with isoproterenol (10 μM) for 24 h in the presence or absence of protocatechuic acid (10 μM) for 6 h. The cells were treated with 10 μM DHE for 30 min in the dark at 37°C in a cell culture incubator and then washed twice with PBS. Images of DHE‐stained cells were captured using fluorescence microscopy (Nikon). Mean fluorescence intensity was measured using ImageJ. Heart tissues from each group were washed with cold PBS and embedded in FSC 22 Frozen Section Media (Leica Biosystems) and frozen in liquid nitrogen. The heart tissue preparations (10 μm thickness) were incubated with 10 μM DHE for 20 min at 25°C in the dark. The heart tissues were washed three times with deionized water. DHE‐stained tissues were analysed using ImageJ.

### 2.15 Statistical analysis

All statistical analyses were performed using GraphPad Prism, version 8 (GraphPad Software). The means among three or more groups were compared using one‐way analysis of variance, followed by the Bonferroni post hoc test.

## 3 RESULTS

### 3.1 Protocatechuic acid alleviates isoproterenol‐induced cardiac hypertrophy in mice

We evaluated the effects of three doses (25, 40 or 80 mg/kg body weight/day) of isoproterenol infusion on C57BL/6NTac male mice. The most efficient isoproterenol dose to establish this heart failure model was 80 mg/kg body weight/day (treatment for 2 weeks; Figure S1). Even at this high concentration, the mice did not die. Isoproterenol treatment increased the end‐diastolic interventricular septal thickness (IVSd) and the end‐diastolic left ventricular posterior wall thickness (LVPWd) and it decreased the left ventricular diameter (Figure S2). These findings indicate that cardiac remodelling is initiated on the fifth day of isoproterenol infusion.

**FIGURE 1:**
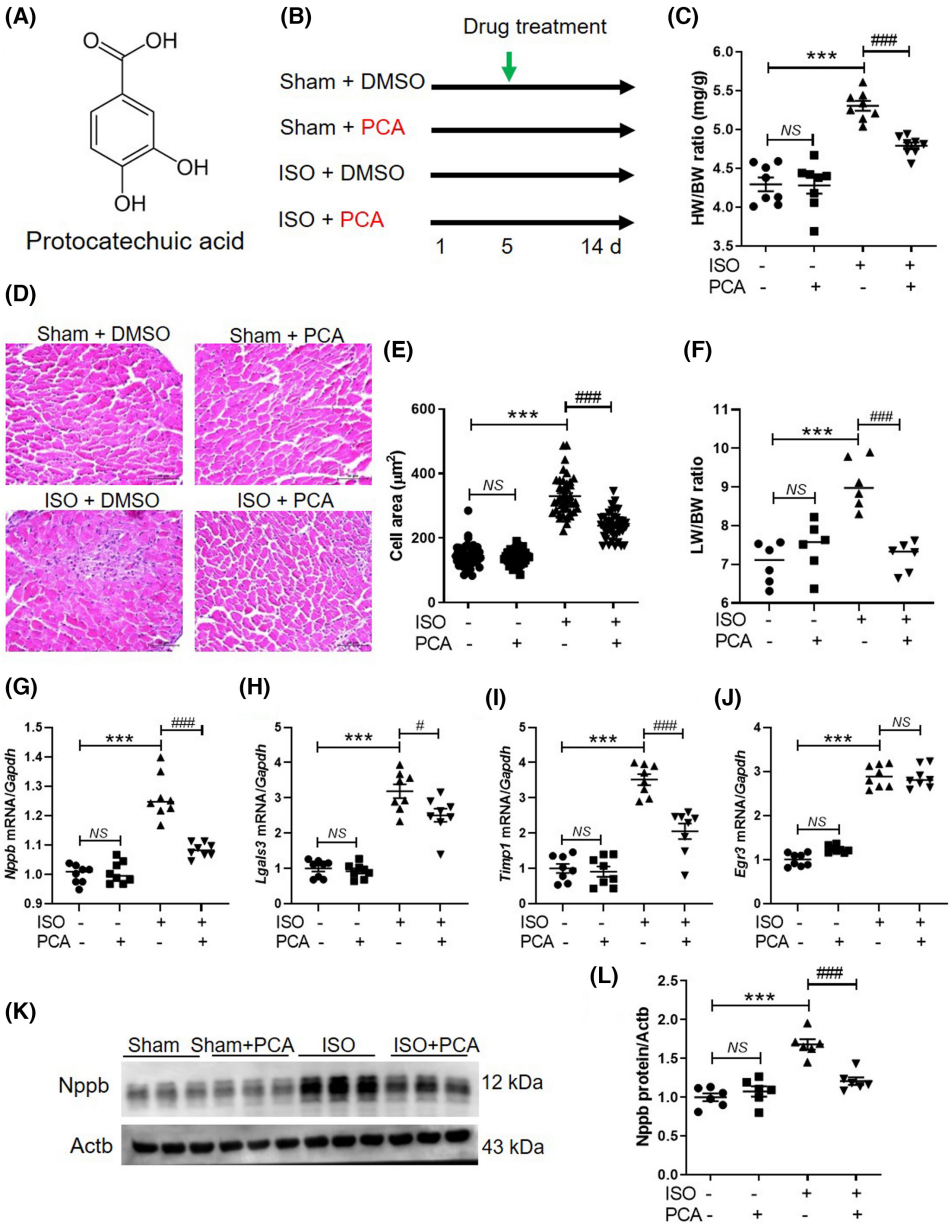
Protocatechuic acid alleviates isoproterenol‐induced cardiac hypertrophy in mice. (A) Chemical structure of protocatechuic acid. (B) Experimental design to evaluate the preventive effect of protocatechuic acid against isoproterenol‐induced heart failure in C57BL/6NTac male mice. (C) Heart‐weight‐to‐body‐weight ratio in different experimental groups (*n* = 8 per group). ***p < 0.001; ^###^ *p* < 0.001. (D, E) Representative images and cell areas of haematoxylin and eosin‐stained cardiac tissues. Scale bar = 50 μm. ***p < 0.001; ^###^ *p* < 0.001. (F) Wet‐lung‐weight‐to‐body‐weight ratio in different experimental groups (*n* = 6 per group). (G–J) The *Nppb*, *Lgals3*, *Timp1* and *Egr3* mRNA levels were determined using qRT‐PCR. ***p < 0.001; ^#^ *p* < 0.05, ^##^ *p* < 0.01, ^###^ *p* < 0.001. (K, L) The Nppb levels were determined using Western blotting (*n* = 6 per group). ***p < 0.001; ^###^ *p* < 0.001. Data are reported as the mean ± standard error of mean. Statistics: one‐way analysis of variance, followed by Bonferroni post hoc tests. DMSO, dimethyl sulfoxide; ISO, isoproterenol; PCA, protocatechuic acid.

For further investigation of whether protocatechuic acid prevents the transition from cardiac hypertrophy to heart failure, intramuscular protocatechuic acid was administered to C57BL/6NTac mice after they had received 5 days of isoproterenol infusion (Figure 1A,B). Protocatechuic acid administration suppressed the isoproterenol‐induced upregulation of the heart‐weight‐to‐body‐weight ratio in C57BL/6NTac mice (Figure 1C) such that this value was not markedly different between the sham + DMSO and sham + protocatechuic acid groups. Haematoxylin and eosin staining revealed that protocatechuic acid decreased cardiomyocyte size in isoproterenol‐treated mice (Figure 1D,E). To further evaluate whether isoproterenol administration induces heart failure, wet lungs were weighed. The wet lung‐weight‐to‐body‐weight ratio was increased in the isoproterenol group, and it was reduced in the protocatechuic acid–treated group (Figure 1F). Additionally, protocatechuic acid decreased the isoproterenol‐induced upregulation of *Nppb*, *Lgals3* and *Timp1* but did not alter *Egr3* mRNA levels (Figure 1G–J). Western blotting analysis revealed that the Nppb levels in the isoproterenol + protocatechuic acid group were downregulated compared with those in the isoproterenol + DMSO group (Figure 1K,L).

### 3.2 Protocatechuic acid alleviates pathological cardiac remodelling and improves cardiac function in this isoproterenol‐induced heart failure model

**FIGURE 2:**
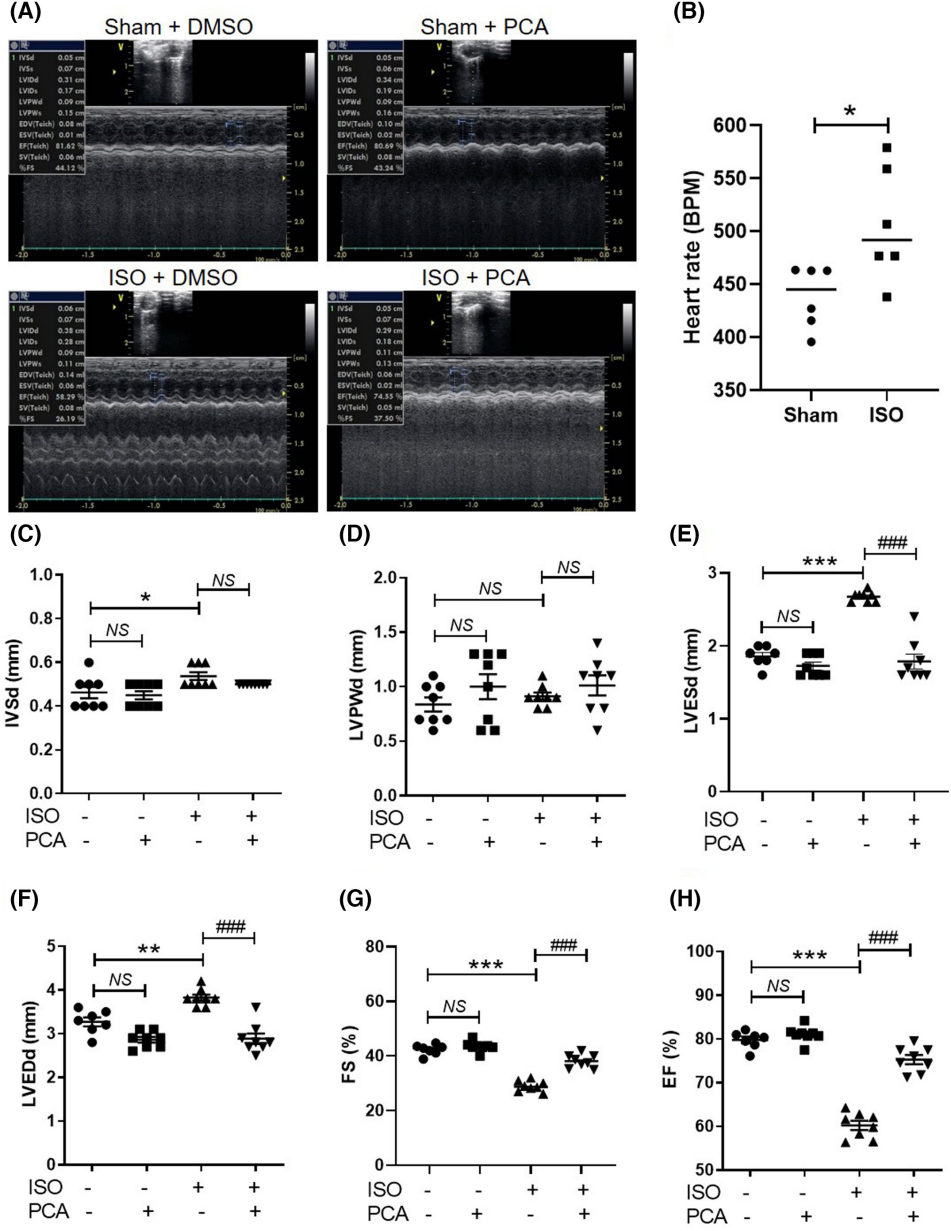
Protocatechuic acid alleviates isoproterenol‐induced pathological cardiac remodelling and improves cardiac functions in mice. (A) Representative M‐mode echocardiographic images from C57BL/6NTac mice. (B) Heart rate in sham and isoproterenol‐infused mice. (C–H) Echocardiographic parameters in mice (*n* = 8 per group). *p < 0.05, **p < 0.01, ***p < 0.001; ^###^ *p* < 0.001. IVSd (mm), end‐diastolic interventricular septum thickness; LVPWd (mm), end‐diastolic left ventricular posterior wall thickness; LVESd (mm), left ventricular end‐systolic diameter; LVEDd (mm), left ventricular end‐diastolic diameter; DMSO, dimethyl sulfoxide; EF, ejection fraction (%); FS, fractional shortening (%); ISO, isoproterenol; PCA, protocatechuic acid.

Treatment with isoproterenol (80 mg/kg body weight/day) resulted in heart failure, which was characterized by an enlarged left ventricular luminal diameter and decreased fractional shortening (Figure 2A). Heart rate was increased in the isoproterenol group compared with the sham group (Figure 2B). The 14 days of isoproterenol infusion slightly increased the IVSd but not the LVPWd in C57BL/6NTac mice (Figure 2C,D). Protocatechuic acid treatment decreased the isoproterenol‐induced increase in left ventricular end‐systolic diameter and left ventricular end‐diastolic diameter to the diameters observed in the sham + DMSO group (Figure 2E,F). The fractional shortening and ejection fraction in the isoproterenol + DMSO group were lower than those in the sham + DMSO group (Figure 2G,H). However, the fractional shortening and ejection fraction in the isoproterenol + protocatechuic acid group were significantly increased compared with those in the isoproterenol + DMSO group.

### 3.3 Protocatechuic acid attenuates cardiac fibrosis in isoproterenol‐treated mice

**FIGURE 3:**
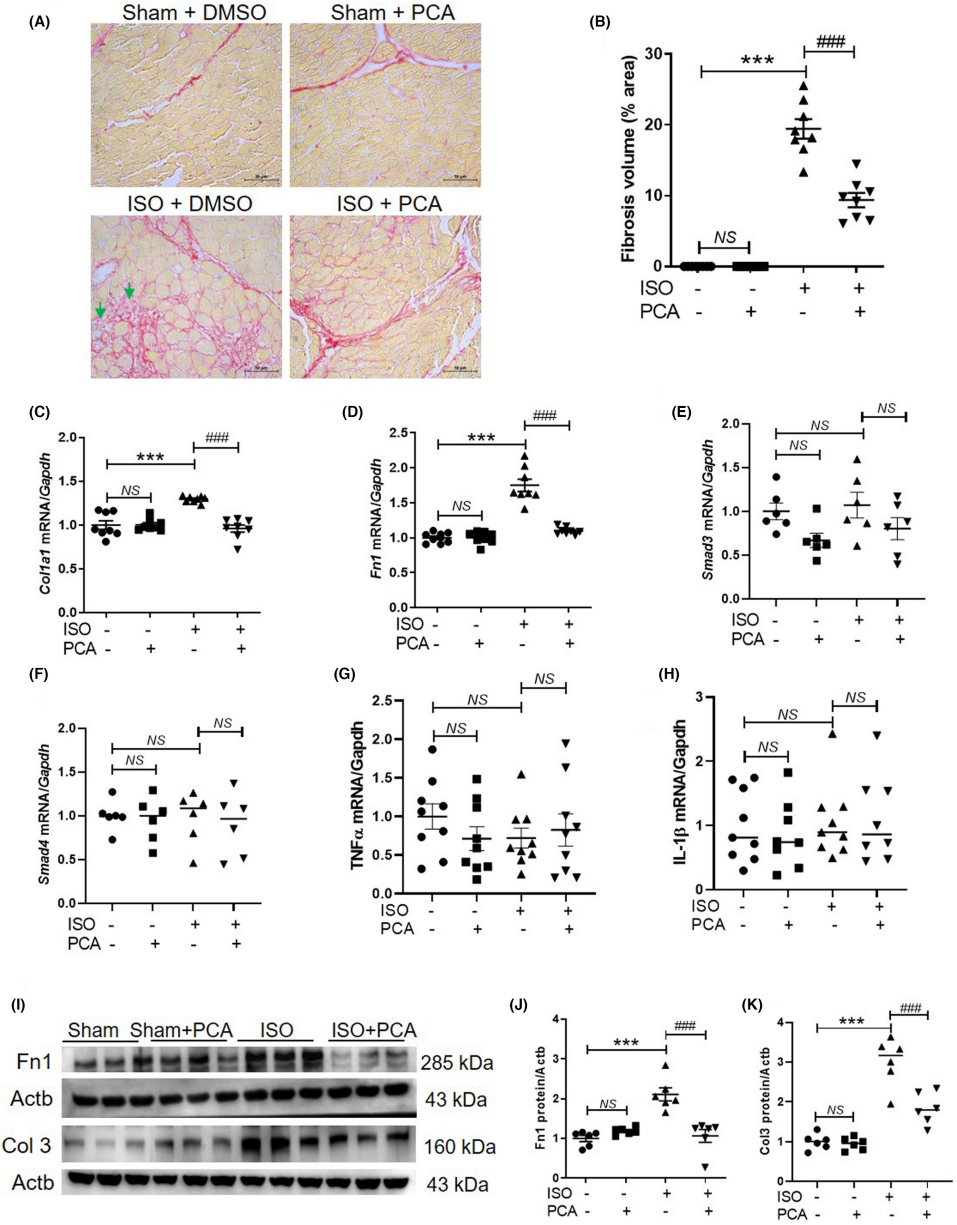
Protocatechuic acid treatment alleviates isoproterenol‐induced cardiac fibrosis in mice. (A) Representative images of picrosirius red–stained cardiac tissues of different groups. Collagen deposition (pink). Green arrows represent isoproterenol‐induced collagen deposition. Scale bar = 50 μm. (B) Quantification of fibrosis (*n* = 8 per group). ***p < 0.001; ^###^ *p* < 0.001. (C–H) The mRNA levels of *Col1a1*, *Fn1*, *Smad3*, *Smad4*, *TNFα*, and *IL‐1β* were quantified and normalized to those of *Gapdh*. ***p < 0.001; ^###^ *p* < 0.001. (I–K) Representative immunoblot images and quantification of the Fn1 and Col3 band intensities in cardiac tissues. Actb was used as a loading control and was the band detected on the same blot used in Figure 1K. ***p < 0.001; ^###^ *p* < 0.001. Data are reported as the mean ± standard error of mean. Statistics: one‐way analysis of variance, followed by Bonferroni post hoc tests. DMSO, dimethyl sulfoxide; ISO, isoproterenol; PCA, protocatechuic acid.

This animal model of heart failure exhibited cardiac fibrosis. Picrosirius red staining revealed that cardiac collagen deposition (pink‐stained areas) in the isoproterenol + DMSO group was greater than that in the sham + DMSO group (Figure 3A). However, protocatechuic acid significantly alleviated isoproterenol‐induced cardiac fibrosis (Figure 3B). Additionally, protocatechuic acid suppressed the isoproterenol‐induced upregulation of cardiac *Col1a1* and *Fn1* mRNA levels (Figure 3C,D). The TGF‐β‐Smads signalling pathway is involved in fibrosis and cardiogenesis.^29^ We examined whether Smad3 and Smad4 can change in this isoproterenol‐induced heart failure model. Analysis with qRT‐PCR showed no differences in the Smad3 and Smad4 mRNA levels between the four groups (Figure 3E,F). Fibrosis is affected by chronic inflammatory processes.^30^ We investigated relevant inflammation‐related gene expression. TNFα and IL‐1β mRNA levels were not altered in heart tissues treated with protocatechuic acid or isoproterenol (Figure 3G,H). Western blotting using anti‐fibronectin and anti‐Col 3 antibodies showed similar effects (Figure 3I–K).

### 3.4 Protocatechuic acid suppresses several signalling pathway–related genes in isoproterenol‐treated mice

**FIGURE 4:**
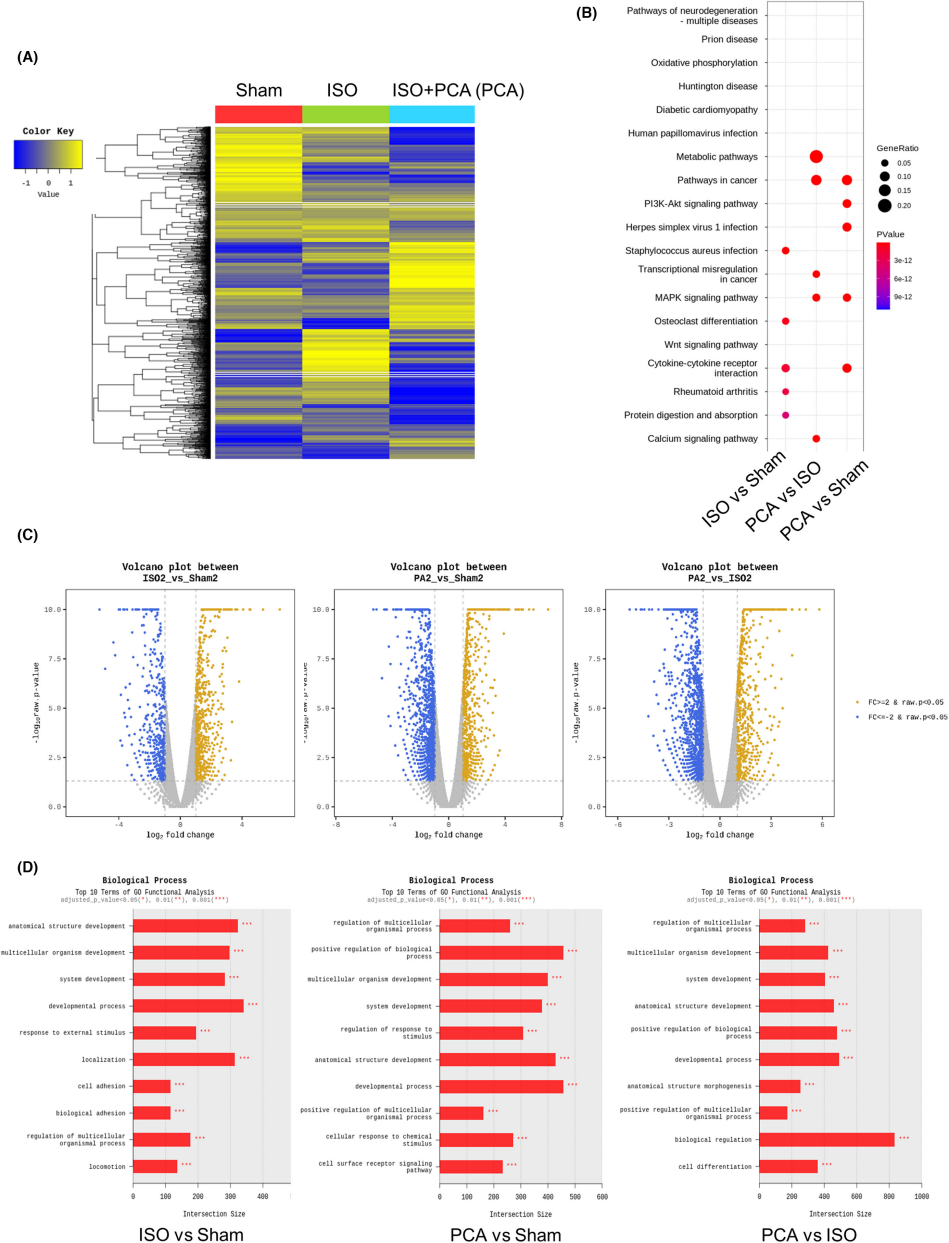
Transcriptome analyses after treatment with protocatechuic acid in an isoproterenol‐induced heart failure model. (A) Heat map of DEGs in the hearts of mice in the sham (Sham), isoproterenol (ISO), isoproterenol + protocatechuic acid (PCA) groups. The colour bar indicates relative expression, with upregulated genes in yellow and downregulated genes in blue. (B) KEGG pathway analysis showing representative pathways between the two groups. (C) Volcano plots of RNA‐seq data obtained from three different combinations. Blue, downregulated genes; yellow, upregulated genes. (D) Top 10 terms in the functional analysis of biological processes among the three groups.

To investigate the genes that play crucial roles in protocatechuic acid–mediated suppression of heart failure, whole ventricular tissues from the sham + DMSO, isoproterenol + DMSO and isoproterenol + protocatechuic acid groups were subjected to RNA sequencing. By analysing differentially expressed genes (DEGs) obtained using gene expression values through mouse transcriptome sequence analysis, a heat map was used to visualize the degree of similarity in the expression patterns of the three groups (Figure 4A). Considering an indicative fold change of >2 and *p*‐values indicating statistical significance, DEGs were found among the three groups: between the sham + DMSO and isoproterenol + DMSO groups, there were 709 upregulated and 671 downregulated genes; between the sham + DMSO and isoproterenol + protocatechuic acid groups, there were 803 upregulated and 1140 downregulated genes; and between the isoproterenol + DMSO and isoproterenol + protocatechuic acid groups, there were 775 upregulated and 1230 downregulated genes. Significant gene lists were subjected to gene‐set enrichment analysis. Kyoto encyclopedia of genes and genomes (KEGG) analysis revealed that DEGs between the isoproterenol + DMSO and isoproterenol + protocatechuic acid groups were enriched in the pathways including metabolic pathways, pathways involved in cancer, transcriptional dysregulation in cancer, the MAPK signalling pathway and calcium signalling pathways (Figure 4B). The volcano plots show the visualized DEGs, the log2 fold change and the −log 10 *p*‐values between the corresponding comparison sets (Figure 4C). Through gene ontology functional analysis, several biological processes were revealed among different combination sets. Common biological processes included regulation of multicellular organismal processes, multicellular organism development, system development, anatomical structure morphogenesis and developmental processes (Figure 4D).

**FIGURE 5:**
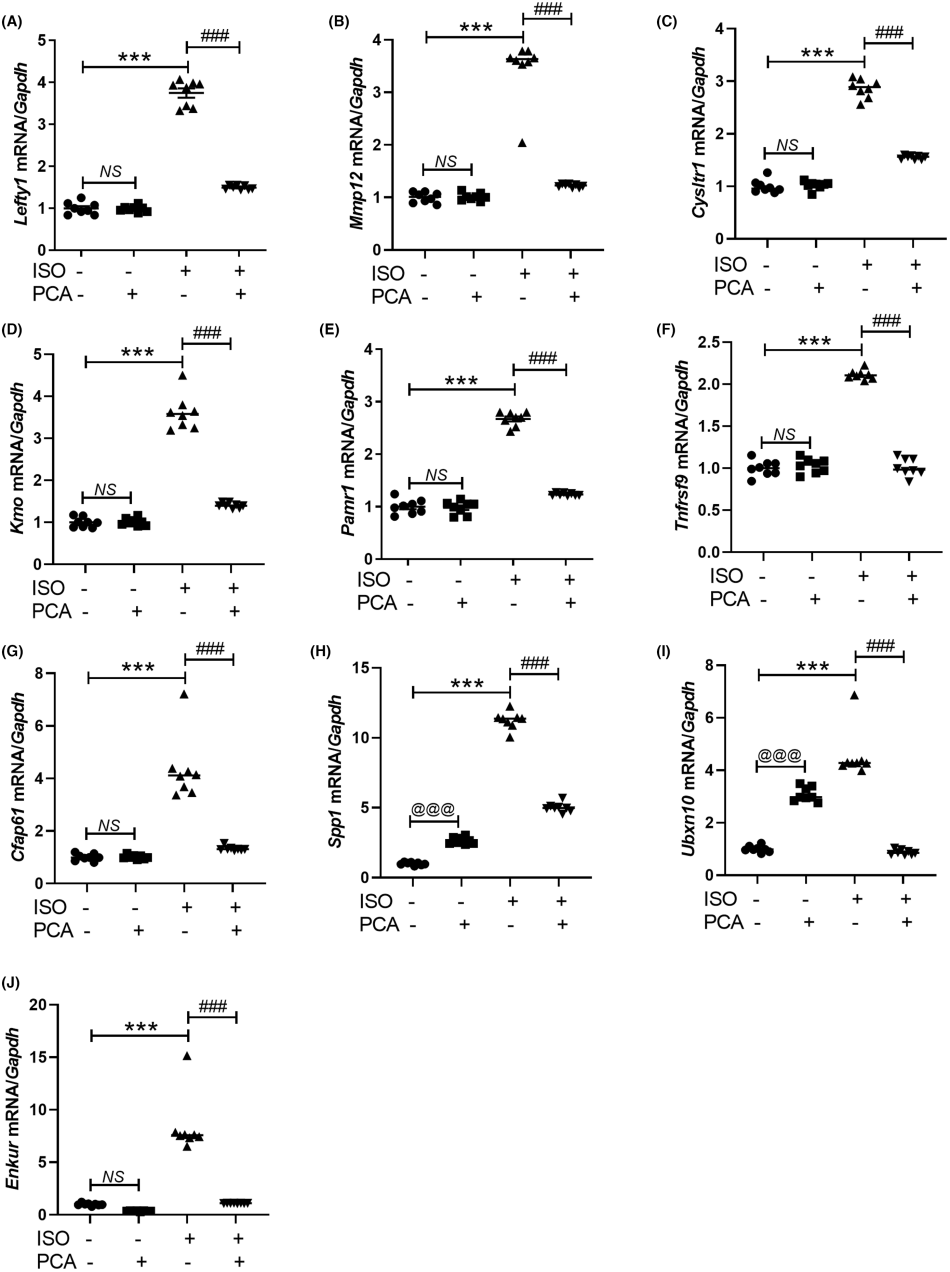
Protocatechuic acid downregulates the expression levels of 10 candidate genes selected from RNA sequencing data in an isoproterenol‐induced heart failure model. (A–J) Ten candidate genes were selected from the RNA sequencing analysis of different groups. The mRNA levels of *Lefty1*, *Mmp12*, *Cysltr1*, *Kmo*, *Pamr1*, *Tnfrsf9*, *Cfap61*, *Spp1*, *Ubxn10* and *Enkur* were determined using qRT‐PCR. ***p < 0.001; ^###^ *p* < 0.001.

Based on the RNA sequencing results, genes that were upregulated in the isoproterenol group relative to the sham group, as well as genes that were downregulated in the isoproterenol + protocatechuic acid group, were selected as candidates. Protocatechuic acid decreased the isoproterenol‐induced upregulation of 10 genes, and this was verified using qRT‐PCR. The roles of these 10 candidate genes in the pathogenesis of cardiac hypertrophy and heart failure have not been previously reported. Protocatechuic acid significantly mitigated the isoproterenol‐induced upregulation of *Lefty1*, *Mmp12*, *Cysltr1*, *Kmo*, *Pamr1*, *Tnfrsf9*, *Cfap61*, *Spp1*, *Ubxn10* and *Enkur* mRNA expression (Figure 5A–J). The RNA sequencing results were consistent with those of qRT‐PCR.

### 3.5 Downregulation of Kmo alleviates fibrosis and suppresses hypertrophic marker genes in H9c2 cells

The roles of *Cysltr1*, *Kmo* and *Lefty1* in H9c2 cardiomyoblasts were investigated. The expression levels of *Cysltr1*, *Kmo* and *Lefty1* were downregulated upon transfection with si‐Cysltr1, si‐Kmo and si‐Lefty1, respectively (Figure S3A–C). Next, the expression of fibrosis‐related genes was examined. The *Col1a1* mRNA levels were unaltered in si‐Cysltr1–transfected cells (Figure S3D), downregulated in si‐Kmo–transfected cells and upregulated in si‐Lefty1–transfected cells (Figure S3E,F). The expression patterns of *Fn1* mRNA were similar to those of *Col1a1* mRNA (Figure S3G–I). Next, the cardiac hypertrophic marker *Nppb* was investigated, and the *Nppb* mRNA levels were downregulated in si‐Cysltr1–transfected cells and si‐Kmo–transfected cells (Figure S3J,K), but they were upregulated in si‐Lefty1–transfected cells (Figure S3L).

### 3.6 Protocatechuic acid treatment or Kmo knockdown alleviates TGF‐β1–induced collagen and fibronectin expression

**FIGURE 6:**
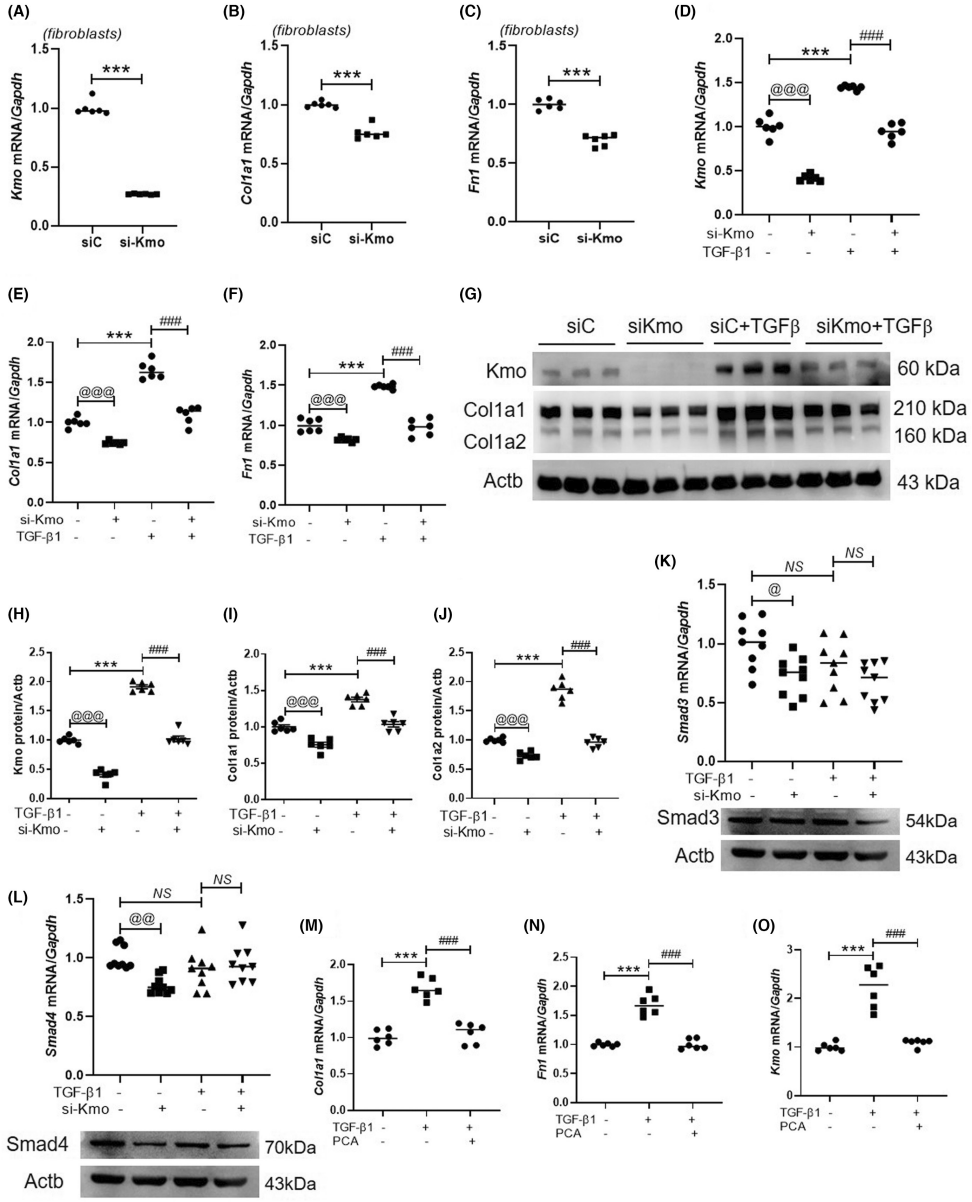
Protocatechuic acid treatment or *Kmo* knockdown alleviates fibrosis in neonatal rat cardiac fibroblasts. (A–C) Primary neonatal rat cardiac fibroblasts were transfected with si‐control or si‐Kmo for 48 h. The mRNA levels of *Kmo*, *Col1a1*, and *Fn1* were assessed using qRT‐PCR (*n* = 6 per group). ***p < 0.001; ^###^ *p* < 0.001. (D–F) The mRNA levels of *Kmo*, *Col1a1* and *Fn1* in transforming growth factor (TGF)‐β1‐treated fibroblasts. ^*@@@*^ *p <* 0.001; ***p < 0.001; ^###^ *p* < 0.001. (G–J) Primary neonatal rat cardiac fibroblasts were transfected with si‐control or si‐Kmo, serum‐starved, and treated with TGF‐β1 for 9 h. (G) Representative immunoblot images. (H–J) Quantification of protein band intensities (*n* = 6 per group). Anti‐Col1a1 antibodies detected the following two isoforms: Col1a1 (210 kDa) and Col1a2 (160 kDa). Actb was used as a loading control. ^*@@@*^ *p* < 0.001; ***p < 0.001; ^###^ *p* < 0.001. (K, L) The mRNA levels of Smad3 and Smad4 in Kmo‐knockdown fibroblasts in the presence or absence of TGF‐β1 (upper panel). Lower panel indicates Smad3 and Smad4 protein levels. Actb was used as a loading control and obtained using the same blot that detected Smad3 and Smad4. (M–O) TGF‐β1–stimulated cardiac fibroblasts were left untreated or were treated with protocatechuic acid. The *Col1a1*, *Fn1* and *Kmo* mRNA levels were determined using qRT‐PCR. ***p < 0.001; ^###^ *p* < 0.001. PCA, protocatechuic acid; siC, si‐control.

The role of Kmo in fibrosis was examined. Primary neonatal rat cardiac fibroblasts were transfected with si‐control and si‐Kmo. The endogenous *Kmo* mRNA levels were downregulated in the si‐Kmo–transfected cells (Figure 6A). Additionally, the *Col1a1* and *Fn1* mRNA levels were downregulated in the si‐Kmo–transfected cells (Figure 6B,C). Moreover, si‐Kmo transfection suppressed the TGF‐β1–induced upregulation of *Kmo*, *Col1a1* and *Fn1* mRNA levels (Figure 6D–F). Western blotting revealed that *Kmo* knockdown attenuated the TGF‐β1–induced upregulation of Kmo, Col1a1 and Col1a2 levels (Figure 6G–J). To further elucidate the association between Kmo and the Smad family in fibrosis, Smad3 and Smad4 expression levels were evaluated in fibroblasts. Knockdown of Kmo decreased the mRNA and protein levels of Smad3 and Smad4. However, there was no change in the expression of these genes in response to TGF‐β1 stimulation (Figure 6K,L). Next, the effects of protocatechuic acid on cardiac fibrosis markers were examined. Protocatechuic acid treatment mitigated the TGF‐β1–induced upregulation of *Col1a1*, *Fn1* and *Kmo* mRNA levels (Figure 6M–O).

### 3.7 Kmo knockdown alleviates isoproterenol‐induced cardiomyocyte hypertrophy in H9c2 cells and primary neonatal rat cardiomyocytes

**FIGURE 7:**
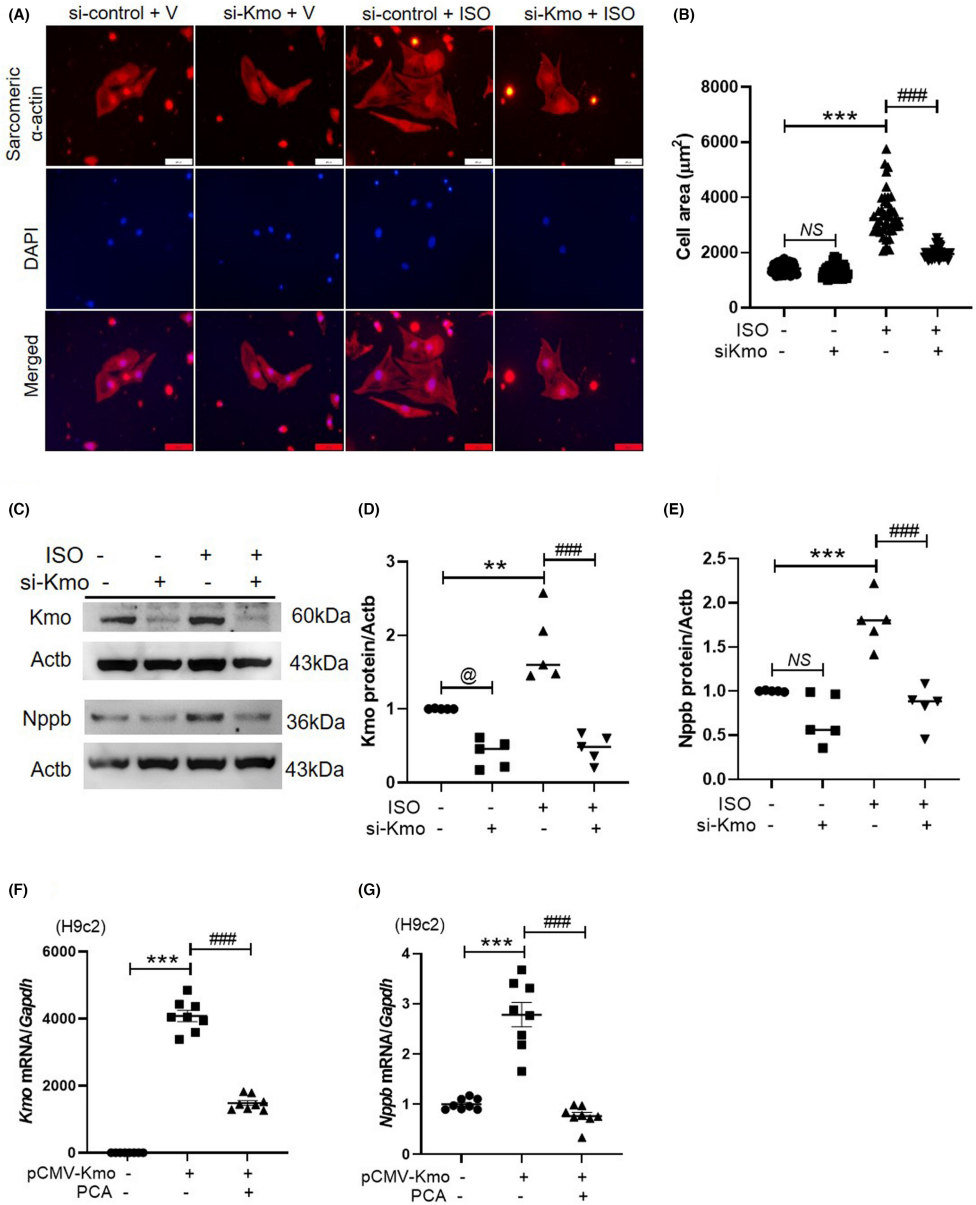
Knockdown of *Kmo* attenuates isoproterenol‐induced cardiomyocyte hypertrophy in primary neonatal cardiomyocytes. (A, B) Rat neonatal cardiomyocytes were transfected with si‐control or si‐Kmo and incubated with vehicle or isoproterenol for 48 h. Representative images of cells stained with anti‐sarcomeric alpha actin (red) and DAPI (blue). Merged images are shown in the lower panels (magnification ×400). Scale bar = 50 μm. (B) Cell areas were measured with a quantitative digital image analysis system (*n* = 41 per group). ***p < 0.001; ^###^ *p* < 0.001. (C–E) si‐Kmo–transfected neonatal cardiomyocytes treated in the presence or absence of isoproterenol were subjected to Western blotting. Nppb (36 kDa) was detected in the glycosylated precursor form. (D, E) Quantification of protein band intensities. **p < 0.01; ^###^ *p* < 0.001. (F, G) H9c2 cells and cardiac fibroblasts were transfected with *pCMV‐SPORT6* or *pCMV‐SPORT6‐mKmo* and treated with vehicle or protocatechuic acid for 9 h. ***p < 0.001; ^###^ *p* < 0.001.

To identify whether Kmo affects cardiomyocyte hypertrophy, the size of si‐control–transfected or si‐Kmo–transfected H9c2 cells that were treated with vehicle or isoproterenol was measured. Alexa Fluor 488 phalloidin staining revealed that *Kmo* knockdown suppressed the isoproterenol‐induced increase in cell area (Figure S4A,B). To further examine the correlation of Kmo with cardiac hypertrophy and fibrosis, Western blotting was performed. *Kmo* knockdown suppressed the isoproterenol‐induced upregulation of Kmo, Nppb, Col1a1 and Col1a2 levels in H9c2 cells (Figure S4C–G). The effect of *Kmo* knockdown was also investigated in primary neonatal rat cardiomyocytes. Sarcomeric alpha actin antibody was used to detect primary cardiomyocytes. The cell area was increased in si‐control–transfected cells in response to isoproterenol treatment. This effect was reduced in si‐Kmo–transfected cells (Figure 7A,B). Western blot analysis showed that the si‐Kmo transfection downregulated endogenous Kmo protein levels (Figure 7C). Isoproterenol‐induced upregulation of Kmo and Nppb protein expression was attenuated by si‐Kmo transfection (Figure 7D,E). Notably, unlike in H9c2 cells, Nppb was detected in a 36 kDa‐glycosylated form in rat primary cardiomyocytes.

### 3.8 Protocatechuic acid attenuates Kmo overexpression–induced hypertrophic marker gene expression in H9c2 cells

To identify whether protocatechuic acid affects Kmo overexpression–induced cardiac hypertrophy, qRT‐PCR was performed. Kmo overexpression increased the endogenous mRNA levels of Kmo in H9c2 cells. The increase was attenuated by protocatechuic acid treatment (Figure 7F). Protocatechuic acid downregulated Kmo overexpression–induced *Nppb* mRNA levels in H9c2 cells (Figure 7G).

### 3.9 Protocatechuic acid or Kmo knockdown attenuates ROS production in vitro and in vivo

**FIGURE 8:**
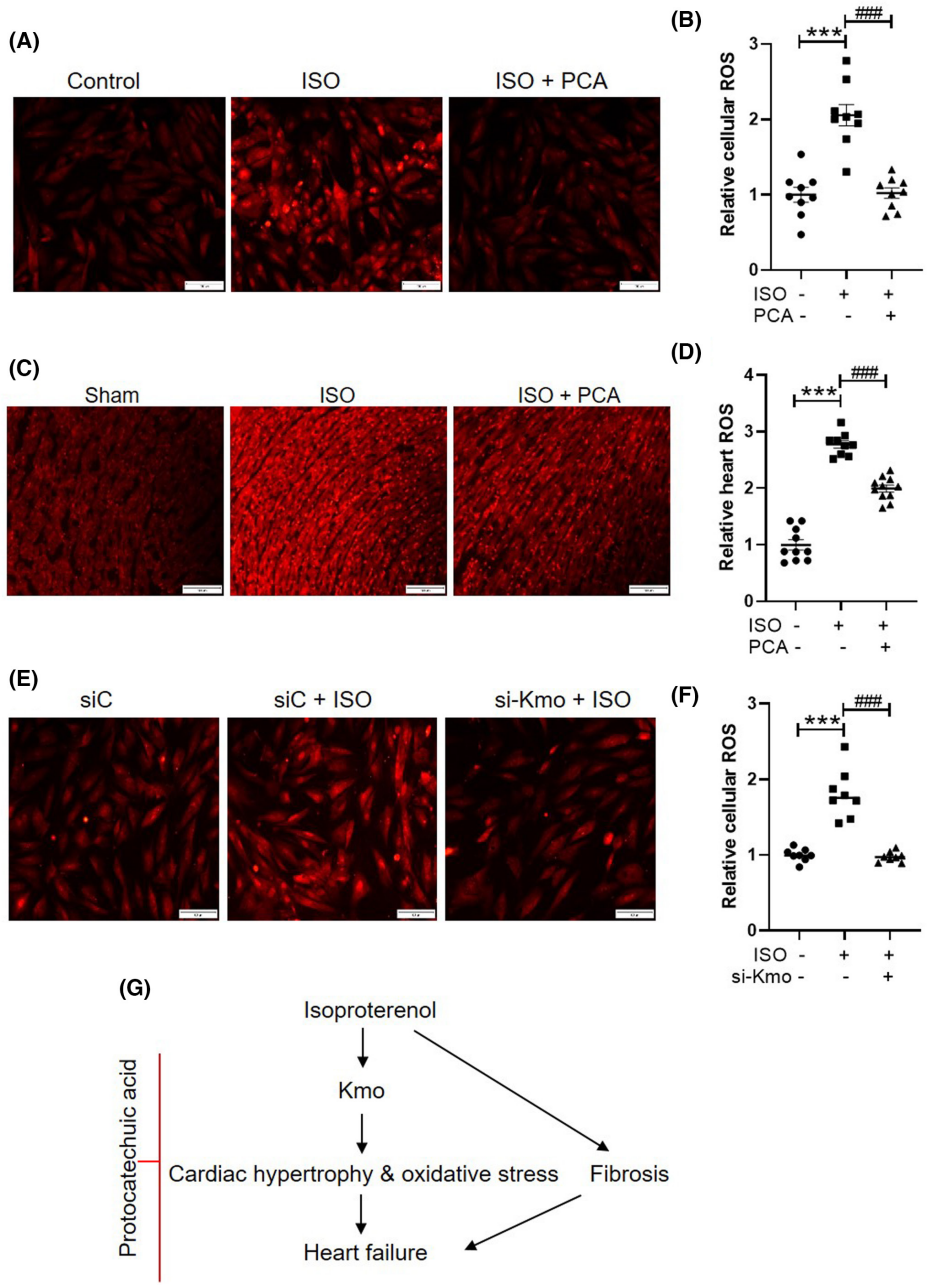
Protocatechuic acid or knockdown of *Kmo* attenuates isoproterenol‐induced ROS production in H9c2 cells or heart tissues. (A, B) Representative DHE‐stained images and ROS levels in isoproterenol‐treated H9c2 cells with or without protocatechuic acid. Scale bar = 100 μm. ***p < 0.001; ^###^ *p* < 0.001. (C, D) Representative DHE‐stained images and ROS levels in isoproterenol‐infused mouse hearts with or without protocatechuic acid. Scale bar = 100 μm. ***p < 0.001; ^###^ *p* < 0.001. (E, F) Representative DHE‐stained images and ROS levels in isoproterenol‐treated si‐Kmo–transfected cells. Scale bar = 100 μm. ***p < 0.001; ^###^ *p* < 0.001. (G) Proposed mechanism of protocatechuic acid–mediated heart failure. Isoproterenol induces heart failure accompanied by cardiac hypertrophy, fibrosis and oxidative stress. Protocatechuic acid treatment suppresses isoproterenol‐induced cardiac hypertrophy, oxidative stress and fibrosis via Kmo regulation.

To determine whether the association between Kmo and cardiac hypertrophy was attributable to oxidative stress, DHE staining was performed in H9c2 cells and heart tissues. Isoproterenol‐treated cells had significantly higher levels of ROS generation than vehicle‐treated cells, and ROS generation was reduced by protocatechuic acid treatment (Figure 8A,B). These observations were similar in isoproterenol‐infused heart tissues with or without protocatechuic acid (Figure 8C,D). To further evaluate whether protocatechuic acid–mediated ROS reduction occurs via Kmo, DHE staining was performed on isoproterenol‐treated Kmo siRNA–transfected cells. Kmo siRNA decreased isoproterenol‐induced ROS production (Figure 8E,F).

## 4 DISCUSSION

This study demonstrated that protocatechuic acid treatment alleviates cardiac hypertrophy, fibrosis, oxidative stress, and dysfunction in an isoproterenol‐induced heart failure mouse model by downregulating Kmo (Figure 8G). The most interesting observation in this study was the restoration of fibrosis‐associated cardiac dysfunction by protocatechuic acid treatment. Protocatechuic acid suppressed the isoproterenol‐induced downregulation of fractional shortening and ejection fraction by decreasing the left ventricular diameter in mice. These findings indicate that protocatechuic acid alleviates heart failure–induced pathological cardiac hypertrophy. Heart failure is accompanied by cardiac hypertrophy and lung oedema. In the present study, the isoproterenol‐induced increase in wet lung mass was attenuated by protocatechuic acid treatment. The analyses of the heart‐weight‐to‐body‐weight ratio, cardiomyocyte cross‐sectional area, and hypertrophic marker genes revealed that protocatechuic acid attenuated cardiac hypertrophy. The mRNA and protein levels of Nppb were downregulated in vivo upon protocatechuic acid treatment. A recent study showed that protocatechuic acid attenuated cardiac hypertrophy in isoproterenol (25 mg/kg body weight/day)–treated Institute of Cancer Research (ICR) male mice,^10^ and this finding is supported by the results of our study. Long‐term isoproterenol infusion induces cardiac hypertrophy and fibrosis comparable to that achieved using the TAC model.^31^ Isoproterenol, a non‐selective β‐adrenergic receptor agonist, contributes to cardiac hypertrophy and fibrosis through β1‐adrenergic signalling activation.^32^, ^33^, ^34^ The TGF‐β1/Smad signalling pathway is implicated in cardiac fibrosis.^35^ Indeed, isoproterenol treatment enhances ColI, Col III, and α‐SMA expression.^8^, ^36^, ^37^ However, in the present study, neither isoproterenol nor TGF‐β1 treatment induced Smad3 and Smad4 expression in heart tissues or cardiac fibroblasts. In contrast with our animal model, the TAC model has been shown to increase Smad3 but not Smad4 protein expression.^38^ Similarly, TGF‐β1 has been shown to upregulate the phospho‐form Smad3 protein but not Smad3 in adult rat cardiac fibroblasts.^39^ Indeed, isoproterenol treatment increases phosphorylated Smad3 protein levels but not Smad3 protein levels in heart and kidney tissues.^8^, ^40^

Picrosirius red staining and analysis of fibrosis‐related genes (*Col1a1* and *Fn1*) revealed that protocatechuic acid attenuated cardiac fibrosis. To identify the target genes of protocatechuic acid, RNA sequencing analysis was performed, and the expression of 10 genes was validated. Among these 10 genes, *Cysltr1* knockdown did not alter the expression of fibrosis‐related genes (*Col1a1* and *Fn1*); however, it downregulated the *Nppb* mRNA levels in cardiac cells. Cysltr1 was implicated in calcium signalling pathways through KEGG analysis. Lefty1, a TGF‐β1 family member, has been shown to attenuate the TGF‐β1‐induced upregulation of Acta2, Fn1, and Vim in NRK‐49F renal fibroblasts.^41^ *Lefty1* knockdown upregulated the expression of fibrosis‐related genes and *Nppb*, indicating that Lefty1 plays a crucial role in alleviating fibrosis and cardiac hypertrophy. Consistent with the findings of this study, adenovirus‐mediated overexpression of Lefty1 has been shown to ameliorate myocardial infarction–induced cardiac fibrosis as well as TGF‐β1‐induced upregulation of Col1 and Col3 in neonatal rat cardiac fibroblasts.^42^ However, KEGG pathway analysis through RNA sequencing showed that the TGF‐β signalling pathway was not related and that the NF‐κB and TNF signalling pathways were related in our animal model. Additionally, KEGG pathway analysis revealed that metabolic pathways—the PI3K‐Akt signalling pathway, the MAPK signalling pathway, and calcium signalling pathways—were involved in the comparative analysis among the three groups (Figure 4B). A recent report^11^ supported our results, suggesting that protocatechuic acid–mediated suppression of cardiac fibrosis and hypertrophy may be linked to the MAPK signalling pathway. Among validated genes from RNA sequencing, *Tnfrsf9* and *Spp1* are associated with cytokine‐cytokine receptor interactions and the PI3K‐Akt signalling pathway, respectively. Moreover, Kmo is associated with metabolic pathways. Considering the RNA sequencing and qRT‐PCR findings, we suggest that several signalling pathways are involved in the protocatechuic acid–mediated alleviation of heart failure. Further detailed mechanistic studies are needed.

*Kmo* knockdown downregulated the expression of fibrosis‐related genes and *Nppb* in cardiac cells and fibroblasts. The results of RNA sequencing analysis of *Kmo* levels in the murine heart were consistent with those of the qRT‐PCR analysis. Knockdown of *Cysltr1* and *Lefty1* did not affect Kmo expression (Figure S5), indicating that *Kmo* is not a downstream gene of *Cysltr1* and *Lefty1* in cardiac cells and fibroblasts. Kmo has been reported to be upregulated in several cancers,^43^ including colorectal cancer and hepatocellular carcinoma. Furthermore, Kmo has been implicated in other diseases, including neurodegenerative diseases.^44^ Kmo inhibition prevents multiorgan failure in acute pancreatitis models.^45^ In this study, the roles of Kmo in cardiac fibroblasts and cardiomyocytes were examined. Consistent with demonstrated effects in cardiac cells, *Kmo* knockdown downregulated *Col1a1* and *Fn1* mRNA levels in neonatal rat cardiac fibroblasts. Additionally, *Kmo* knockdown mitigated the TGF‐β1–induced upregulation of *Col1a1* and *Fn1* mRNA levels in fibroblasts. These findings suggest that Kmo is a novel therapeutic target for cardiac fibrosis. Genetic deletion of Kmo exerted a protective effect against ischemia–reperfusion injury–induced acute kidney injury.^46^ Nonetheless, contradictory results have been reported regarding the role of Kmo in the kidney. Kmo downregulation in people with diabetes is associated with proteinuria.^47^ However, in this study, Kmo downregulation suppressed the isoproterenol‐induced increase in cardiomyocyte size and Nppb expression, indicating that Kmo is involved in cardiac hypertrophy. Considering the upregulated Kmo expression in this heart failure animal model, we can measure the serum levels of Kmo or Kmo metabolites of patients with heart failure and analyse the correlations. Therefore, serum Kmo levels can be used as a biomarker for predicting heart failure similar to other biomarkers, such as NT‐proBNP, troponin, sST2, galectin‐3, and growth differentiation factor‐15.^48^, ^49^

The findings of some recent studies are consistent with those of this study with regard to the therapeutic effects of protocatechuic acid on cardiac fibrosis. Protocatechuic acid attenuates fibrosis in the liver,^20^ heart,^11^ and lungs.^50^ In this study, protocatechuic acid attenuated fibrosis in an isoproterenol‐induced heart failure model and in TGF‐β1–treated cardiac fibroblasts. Chronic exposure to isoproterenol can induce cardiomyocyte damage through upregulation of ROS generation in heart failure.^51^ Indeed, we demonstrated that isoproterenol causes oxidative stress in heart tissue^52^ and cardiomyocytes,^53^ which was attenuated by protocatechuic acid treatment or Kmo downregulation. These findings indicate that Kmo or protocatechuic acid can be a novel target gene and therapeutic agent, respectively, in the inhibition of isoproterenol‐induced cardiac hypertrophy and oxidative stress.

### 4.1 Limitations

*Kmo* is one of the target genes that mediate the therapeutic effects of protocatechuic acid against heart failure. Therefore, this study considered *Kmo* as a candidate gene for alleviating cardiac fibrosis and hypertrophy. However, the functions of other target genes that were modulated by protocatechuic acid have not yet been elucidated. Considering the therapeutic benefits of Kmo inhibitors in various diseases, the findings of this study will be strengthened if future studies demonstrate the alleviation of heart failure following treatment with Kmo inhibitors. Another limitation was the use of neonatal cardiac cells instead of adult cardiac cells to mimic cardiac hypertrophy in vivo. Neonatal cardiomyocyte isolation is much easier (and is associated with higher cell yields) than adult cardiomyocyte isolation. A major advantage of neonatal cardiomyocytes is their spontaneously beating phenotype that precludes the need to administer agents to induce contractions.^54^ Even though isoproterenol infusion readily causes cardiac hypertrophy and fibrosis, the use of high doses of isoproterenol enhances the associated myocardial impairments. Therefore, the use of animal models for left coronary ligation or TAC can overcome the limitations of the isoproterenol model.

## 5 CONCLUSIONS

Protocatechuic acid alleviated cardiac dysfunction, fibrosis and oxidative stress by downregulating Kmo in vivo and in vitro in an isoproterenol‐induced heart failure model. The therapeutic effects of protocatechuic acid against heart failure were mediated through Kmo. The findings of this study suggest that the protocatechuic acid–mediated downregulation of Kmo is a potential therapeutic strategy for preventing heart failure.

## AUTHOR CONTRIBUTIONS

**Liyan Bai:** Data curation (lead); formal analysis (lead); investigation (lead); methodology (lead); writing – review and editing (equal). **Xiongyi Han:** Conceptualization (equal); data curation (equal); investigation (equal); methodology (equal); writing – review and editing (equal). **Hae Jin Kee:** Conceptualization (lead); data curation (supporting); funding acquisition (equal); investigation (lead); project administration (equal); resources (lead); supervision (lead); writing – original draft (lead). **Xiaonan He:** Writing – review and editing (equal). **Seong Hoon Kim:** Data curation (supporting); methodology (supporting). **Mi Jin Jeon:** Data curation (equal); formal analysis (equal). **Hongyan Zhou:** Data curation (equal); formal analysis (equal). **Seong Min Jeong:** Formal analysis (equal). **Seung‐Jung Kee:** Resources (equal); writing – review and editing (equal). **Myung Ho Jeong:** Funding acquisition (equal); supervision (equal); writing – review and editing (equal).

## FUNDING INFORMATION

This study was supported by the Basic Science Research Program through the National Research Foundation of Korea (NRF), funded by the Ministry of Education (grant numbers NRF‐2021R1I1A3045431 and 2022R1A2C1004051).

## CONFLICT OF INTEREST STATEMENT

None of the authors have any conflicts of interest to declare.

## Supporting information


Figure S1.

Figure S2.

Figure S3.

Figure S4.

Figure S5.

Data S1.

## Data Availability

The data used to support the findings of this research are available from the corresponding author upon request.
