# Supplementary figures and images for: Protocatechuic acid prevents isoproterenol‐induced heart failure in mice by downregulating kynurenine‐3‐monooxygenase
Source: J Cell Mol Med. 2023 Jul 22;27(16):2290–307. doi: 10.1111/jcmm.17869 (PMC10424289; doi:10.1111/jcmm.17869)

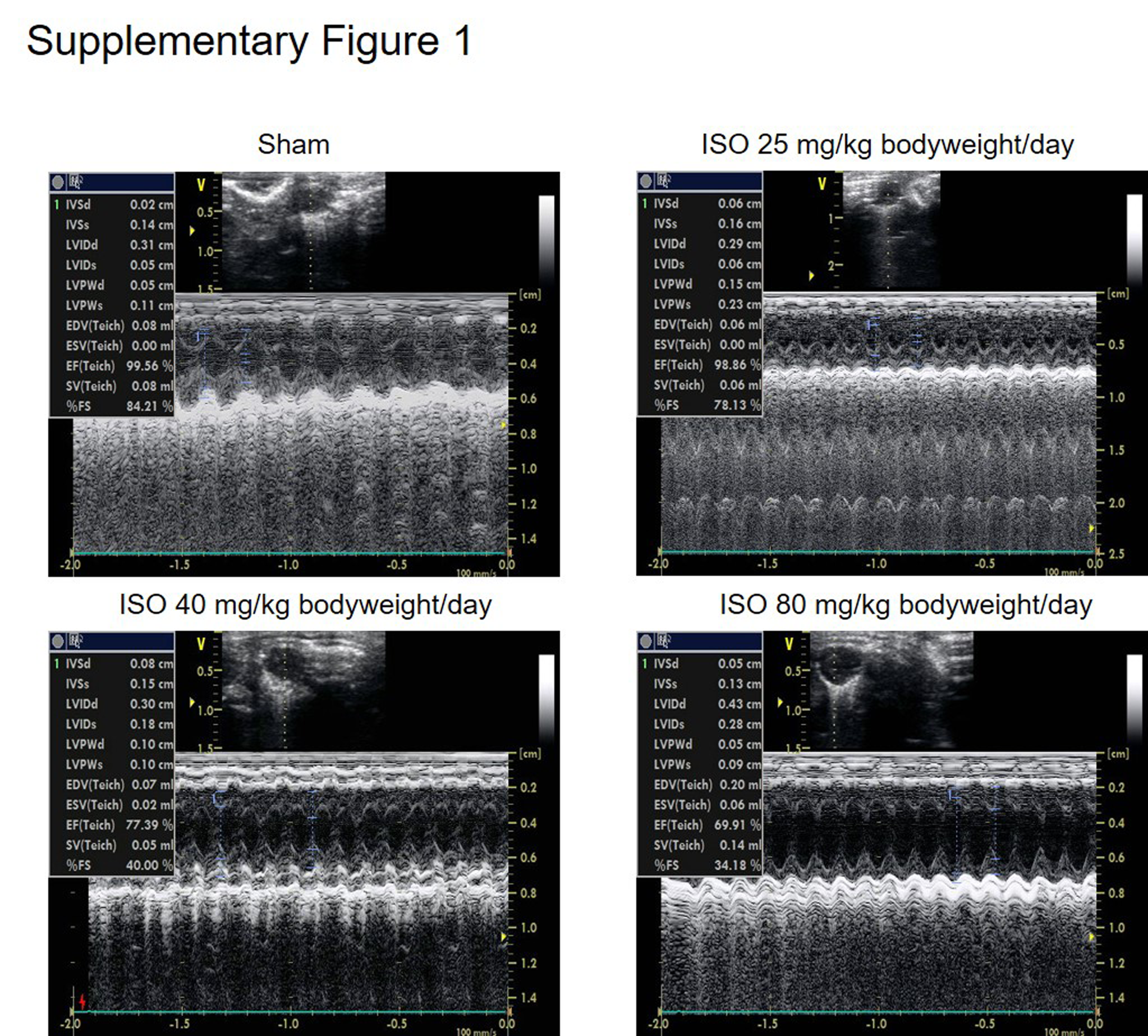

Supplement: Supplementary file 1 — Figure S1. [file JCMM-27-2290-s001.tif]

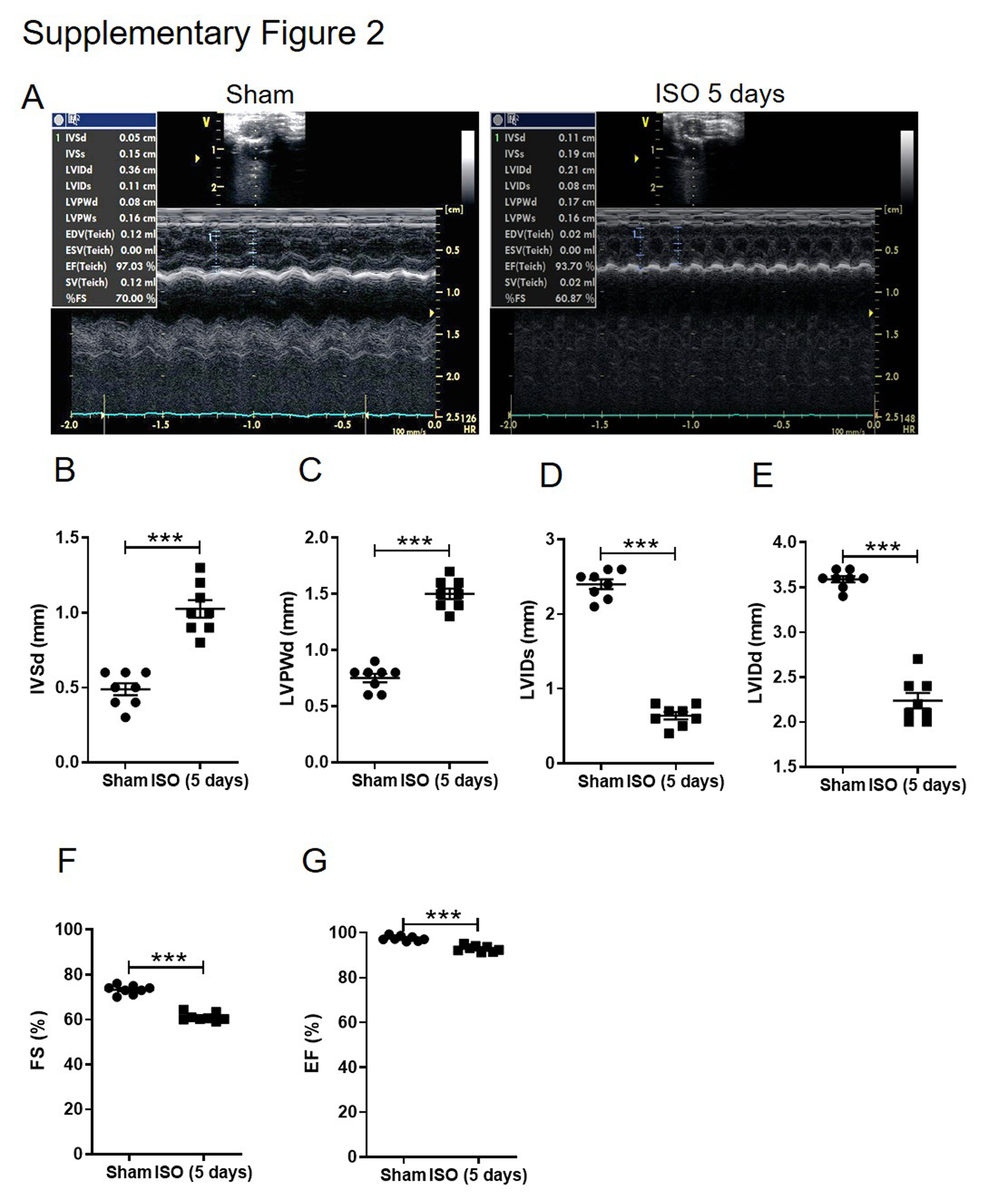

Supplement: Supplementary file 2 — Figure S2. [file JCMM-27-2290-s002.tif]

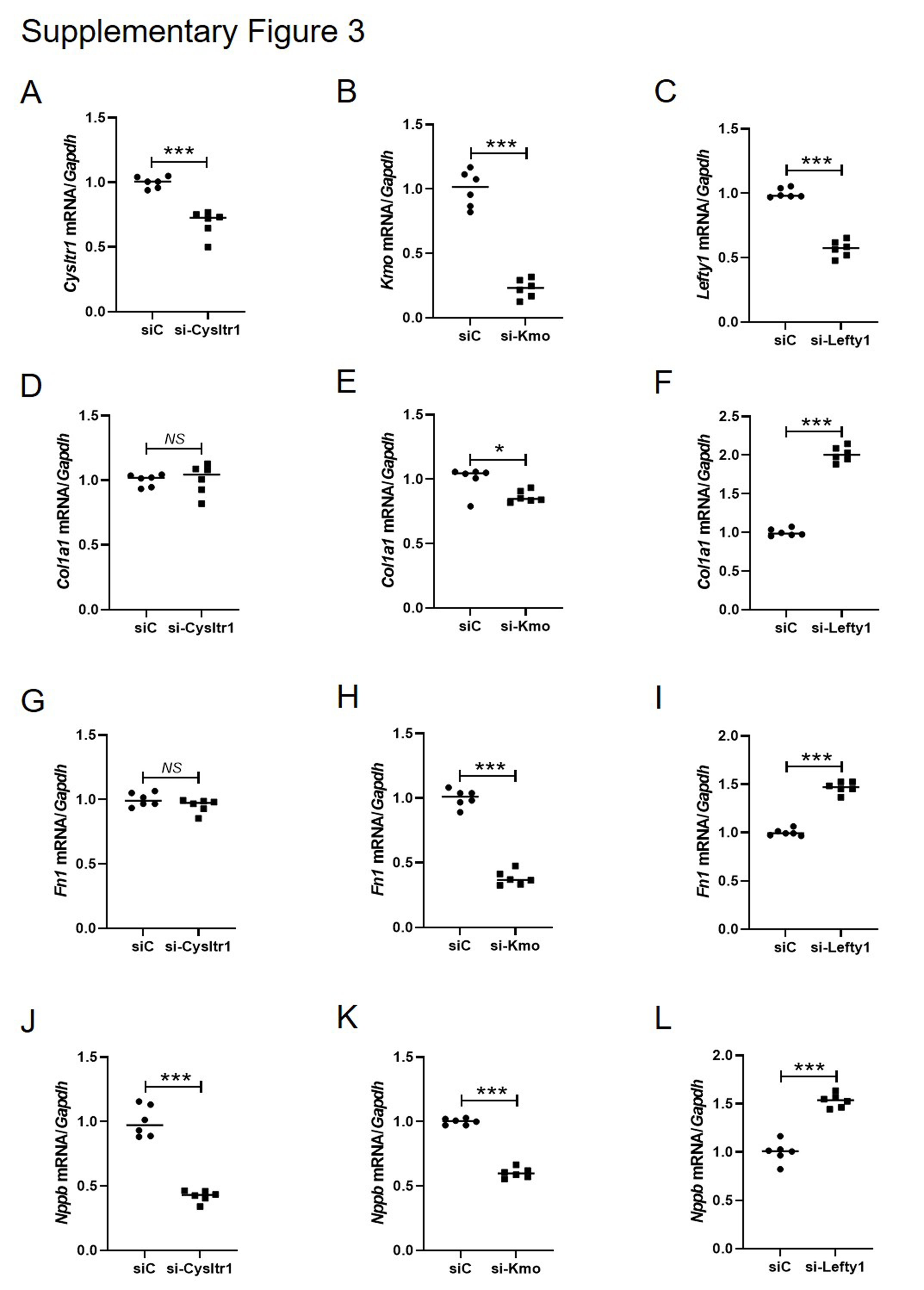

Supplement: Supplementary file 3 — Figure S3. [file JCMM-27-2290-s004.tif]

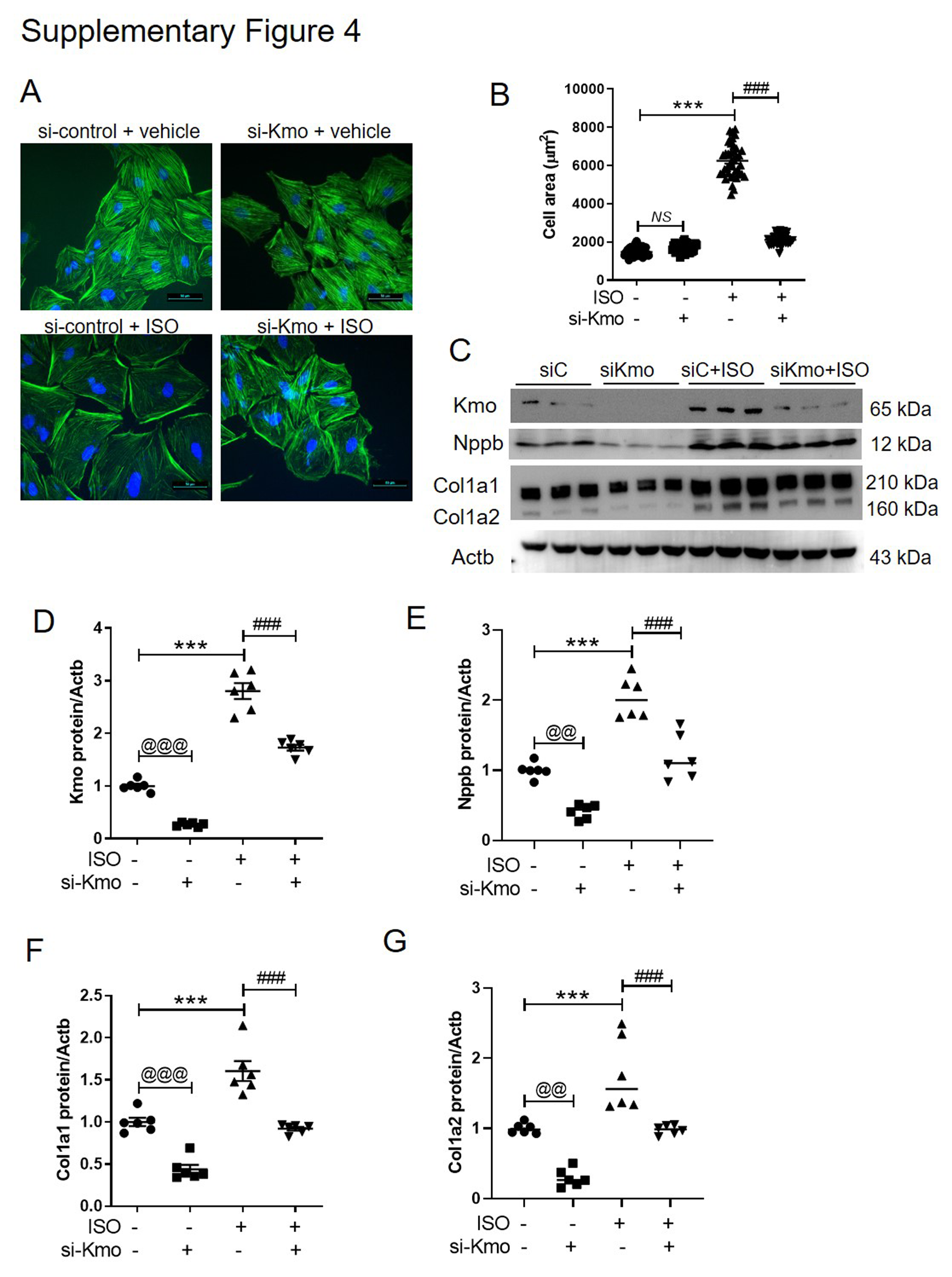

Supplement: Supplementary file 4 — Figure S4. [file JCMM-27-2290-s005.tif]

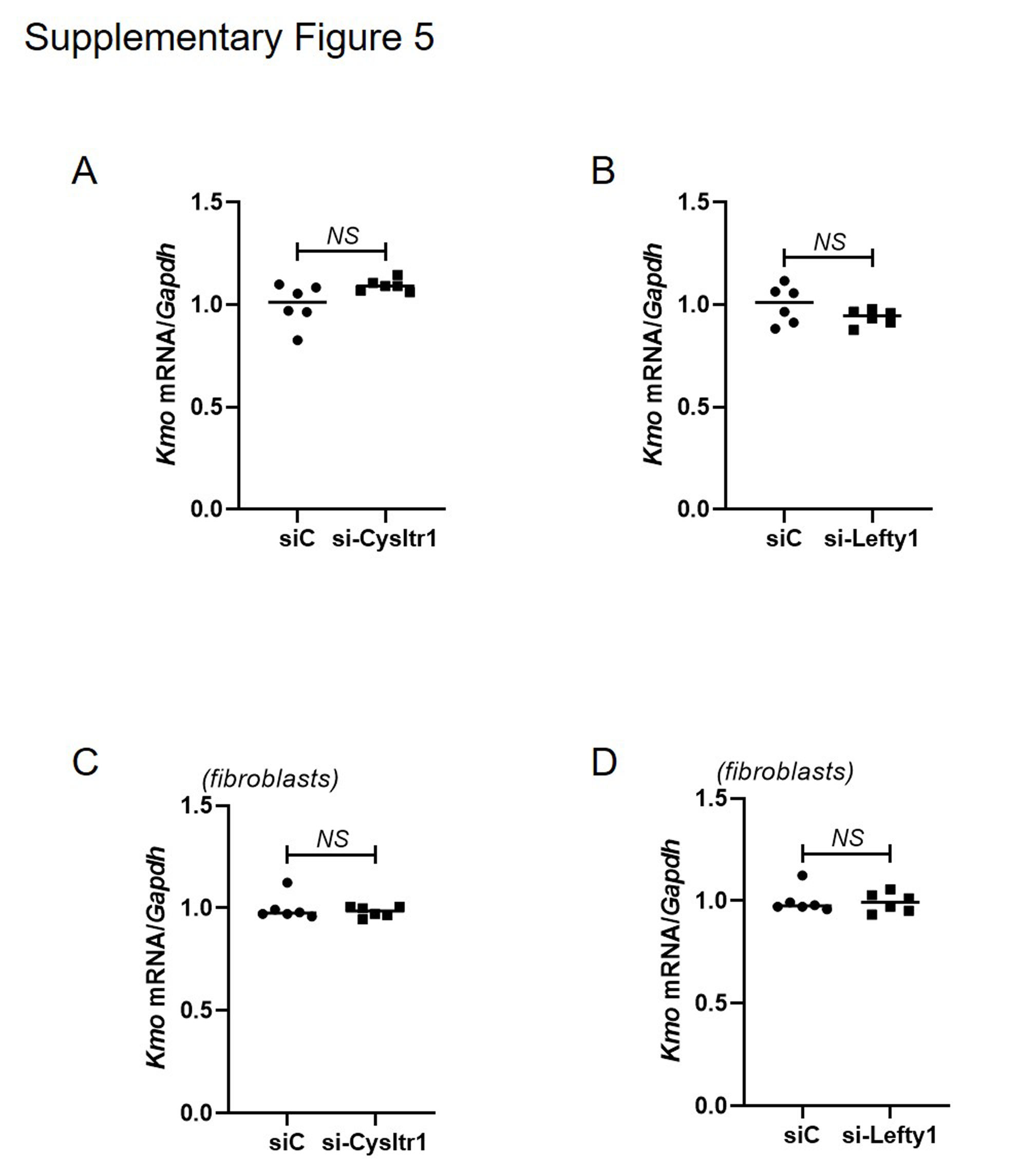

Supplement: Supplementary file 5 — Figure S5. [file JCMM-27-2290-s003.tif]
