# Supplementary material for: Protocatechuic acid prevents isoproterenol‐induced heart failure in mice by downregulating kynurenine‐3‐monooxygenase
Source: J Cell Mol Med. 2023 Jul 22;27(16):2290–307. doi: 10.1111/jcmm.17869 (PMC10424289; doi:10.1111/jcmm.17869)
Supplement: Supplementary file 6 — Data S1. [file JCMM-27-2290-s006.doc]

**SUPPORTING INFORMATION**

# Protocatechuic Acid Prevents Isoproterenol-induced Heart Failure in Mice by Downregulating Kynurenine-3-monooxygenase

Liyan Bai^1,2,3,#^, Xiongyi Han^1,2,4#^, Hae Jin Kee^1,2,*^, Xiaonan He^3^, Seong Hoon Kim^5^, Mi Jin Jeon^1,2^, Hongyan Zhou^1,2^, Seong Min Jeong^1,2^, Seung-Jung Kee^6^, and Myung Ho Jeong^1,2,7,*^

^1^Heart Research Center of Chonnam National University Hospital, Gwangju, Republic of Korea

^2^Hypertension Heart Failure Research Center, Chonnam National University Hospital, Gwangju, Republic of Korea

^3^Emergency Critical Center, Beijing Anzhen Hospital, Capital Medical University, Beijing, People’s Republic of China

^4^Aerospace Center Hospital, Peking University Aerospace School of Clinical Medicine, Beijing, Republic of China

^5^Department of Parasitology and Tropical Medicine, Chonnam National University Medical School, Hwasun, Republic of Korea

^6^Department of Laboratory Medicine, Chonnam National University, Medical School and Hospital, Gwangju, Republic of Korea

^7^Department of Cardiology, Chonnam National University Medical School, Gwangju, Republic of Korea

#These authors contributed equally to this work.

*Correspondence to

Hae Jin Kee, PhD

Heart Research Center of Chonnam National University Hospital, 42 Jebong-ro, Dong-gu, Gwangju 61469, Republic of Korea; Tel.: +82-62-220-5823; Fax: +82-62-228-4227; E-mail: [sshjkee@empas.com](mailto:sshjkee@empas.com)

Myung Ho Jeong, MD, PhD, FACC, FAHA, FESC, FSCAI, FAPSIC

Director of Heart Research Center Nominated by Korea Ministry of Health and Welfare, Chonnam National University Hospital, 42 Jebong-ro, Dong-gu, Gwangju 61469, Republic of Korea; Tel.: +82-62-220-6243; Fax: +82-62-228-7174; E-mail: myungho@chollian.net

**Supplementary Figures**

**Figure S1. Establishment of an isoproterenol-induced heart failure animal model.**

C57BL/6 male mice were infused with different doses of isoproterenol (0, 25, 40, or 80 mg/kg bodyweight) using an osmotic minipump for 14 days. Representative M-mode echocardiograms are shown. ISO, isoproterenol.

**Figure S2. Isoproterenol infusion for 5 days induces a mild heart failure phenotype in C57BL/6 mice.**

After 5 days of isoproterenol infusion, echocardiographic parameters were examined in the sham and isoproterenol groups. (**A**) Representative M-mode echocardiograms of the sham and isoproterenol groups. (**B**) End-diastolic interventricular septum thickness (IVSd). (**C**) Left ventricular posterior wall thickness LVPWd. (**D**) Left ventricular internal diameter at systole LVIDs. (**E**) Left ventricular internal diameter at diastole (LVIDd). (**F**) Fractional shortening (FS). (**G**) Ejection fraction (EF). ****P < 0.001*. ISO, isoproterenol.

**Figure S3. Knockdown of Kmo downregulates fibrosis-related genes and Nppb in H9c2 cells.**

(A–L) H9c2 cells were transfected with si-control, si-Cysltr1, si-Kmo, or si-Lefty1 for 48 h. The mRNA levels of Cysltr1, Kmo, Lefty1, Col1a1, Fn1, and Nppb were determined using qRT-PCR (n = 6 per group). ***p < 0.001; ^###^p < 0.001. siC; si-control

**Figure S4. Knockdown of Kmo attenuates isoproterenol-induced cardiomyocyte hypertrophy and fibrosis in H9c2 cells.**

(A−B) H9c2 cells were transfected with si-control or si-Kmo and incubated with vehicle or isoproterenol (ISO) for 24 h. Representative images of cells stained with Alexa Fluor 488 phalloidin (actin staining) and quantification of the staining intensity (n = 40 per group). Scale bar = 50 μm. ***p < 0.001; ^###^p < 0.001. (C−G) si-Kmo-transfected H9c2 cells treated in the presence or absence of isoproterenol for 9 h were subjected to Western blotting. (D−G) Quantification of protein band intensities. @@@p < 0.001; ***p < 0.001; ^###^p < 0.001.

**Figure S5. Knockdown of *Cysltr1* and *Lefty1* did not affect the expression of *Kmo* in H9c2 and rat neonatal cardiac fibroblasts.**

(**A–B**) H9c2 cells were transfected with si-control, si-Cysltr1, or si-Lefty1. (**C–D**) Rat neonatal cardiac fibroblast cells were transfected with si-control, si-Cysltr1, or si-Lefty1. The *Kmo* mRNA levels were determined using quantitative real-time polymerase chain reaction.
